# PLEKHM2 deficiency induces impaired mitochondrial clearance and elevated ROS levels in human iPSC-derived cardiomyocytes

**DOI:** 10.1038/s41420-024-01907-6

**Published:** 2024-03-15

**Authors:** Jianchao Zhang, Ying Peng, Wanrong Fu, Ruifei Wang, Jinhua Cao, Shuang Li, Xiaoxu Tian, Zhonggen Li, Chongpei Hua, Yafei Zhai, Yangyang Liu, Mengduan Liu, Jihong Sun, Xiaowei Li, Xiaoyan Zhao, Jianzeng Dong

**Affiliations:** 1 Department of Cardiology, The First Affiliated Hospital of Zhengzhou University, Zhengzhou, 450052 China; 2 Henan Key Laboratory of Hereditary Cardiovascular Diseases, Zhengzhou, 450052 China; 3 Department of Cardiology, The Second Affiliated Hospital of Zhengzhou University, Zhengzhou, 450052 China; 4 School of Life Sciences, Zhengzhou University, Zhengzhou, 450001 Henan China; 5 Department of Cardiology, Beijing Anzhen Hospital, Capital Medical University, National Clinical Research Centre for Cardiovascular Diseases, No. 2 Beijing Anzhen Road, Chaoyang District, Beijing, 100029 China

**Keywords:** Genetics, Cardiomyopathies, Cell biology

## Abstract

Pleckstrin homology domain-containing family M member 2 (PLEKHM2) is an essential adaptor for lysosomal trafficking and its homozygous truncation have been reported to cause early onset dilated cardiomyopathy (DCM). However, the molecular mechanism of PLEKHM2 deficiency in DCM pathogenesis and progression is poorly understood. Here, we generated an in vitro model of PLEKHM2 knockout (KO) induced pluripotent stem cell-derived cardiomyocytes (hiPSC-CMs) to elucidate the potential pathogenic mechanism of PLEKHM2-deficient cardiomyopathy. PLEKHM2-KO hiPSC-CMs developed disease phenotypes with reduced contractility and impaired calcium handling. Subsequent RNA sequencing (RNA-seq) analysis revealed altered expression of genes involved in mitochondrial function, autophagy and apoptosis in PLEKHM2-KO hiPSC-CMs. Further molecular experiments confirmed PLEKHM2 deficiency impaired autophagy and resulted in accumulation of damaged mitochondria, which triggered increased reactive oxygen species (ROS) levels and decreased mitochondrial membrane potential (Δψm). Importantly, the elevated ROS levels caused oxidative stress-induced damage to nearby healthy mitochondria, resulting in extensive Δψm destabilization, and ultimately leading to impaired mitochondrial function and myocardial contractility. Moreover, ROS inhibition attenuated oxidative stress-induced mitochondrial damage, thereby partially rescued PLEKHM2 deficiency-induced disease phenotypes. Remarkably, PLEKHM2-WT overexpression restored autophagic flux and rescued mitochondrial function and myocardial contractility in PLEKHM2-KO hiPSC-CMs. Taken together, these results suggested that impaired mitochondrial clearance and increased ROS levels play important roles in PLEKHM2-deficient cardiomyopathy, and PLEKHM2-WT overexpression can improve mitochondrial function and rescue PLEKHM2-deficient cardiomyopathy.

## Introduction

Dilated cardiomyopathy (DCM) is a common and severe form of heart failure that arises from the multifactorial etiology of genetic and environmental factors [1]. Loss-of-function mutations in Pleckstrin homology and RUN domain-containing M2 (*PLEKHM2*) have been implicated as a genetic cause of recessive DCM in previous clinical studies [2, 3]. These studies also revealed that PLEKHM2-deficient patients exhibited early-onset ventricular dilatation, impaired systolic function and severe ventricular arrhythmia [3]. PLEKHM2 is a protein that interacts with the kinesin-1 motor protein, the BORC complex and the Arl8 GTPase to mediate the anterograde transport of lysosomes along microtubules [4]. The bidirectional movement of lysosomes between the perinuclear and peripheral regions ensures the proper distribution and delivery of diverse cellular cargoes [5, 6]. Previous studies using fibroblasts from patients with homozygous truncating mutations in *PLEKHM2* have suggested that PLEKHM2 deficiency cause abnormal lysosomal positioning and impaired autophagic flux [2]. Although these results reveal the importance of PLEKHM2 in maintaining cellular homeostasis and lysosomal function, the exact molecular mechanisms by which PLEKHM2 deficiency contribute to early-onset DCM remain elusive.

Previous studies have reported the important role of PLEKHM2 in patient-derived fibroblasts or HeLa cells [3, 4], but there is still a lack of suitable in vivo or in vitro cardiomyopathy models to reveal the effects of PLEKHM2 deficiency on cardiomyocytes or heart tissues. Human induced pluripotent stem cell-derived cardiomyocytes (hiPSC-CMs) can recapitulate key features of cardiomyocytes, such as contractility, calcium handling, and electrophysiology, and thus offer an in vitro platform to model diverse cardiomyopathies [7]. Moreover, hiPSC-CMs provide a more human-relevant system than traditional animal models, thereby minimizing the potential of species-specific differences in disease manifestation and treatment response [8]. Importantly, the integration of hiPSC and CRISPR-Cas9 genome editing technology has significantly improved the capacity to model human diseases by introducing specific genetic alterations [9]. Regarding PLEKHM2-deficient cardiomyopathy, Muhammad et al. [2] and Atkins et al. [3] respectively reported two cases of *PLEKHM2* loss-of-function mutations leading to recessive DCM, one with a homozygous frameshift mutation and one with a compound heterozygous mutation consisting of a frameshift and a splice mutation. In these cases, *PLEKHM2* underwent a biallelic frameshift or splice mutation, resulting in a deficiency of the PLEKHM2 protein. Thus, we established an in vitro model of PLEKHM2-KO hiPSC-CMs by leveraging the CRISPR/Cas9 gene editing tool to knockout (KO) the *PLEKHM2* gene in healthy donor-derived hiPSCs.

In this study, we demonstrated that PLEKHM2-KO hiPSC-CMs developed disease phenotypes with reduced myocardial contractility and abnormal calcium handling. Furthermore, we uncovered that impaired mitochondrial clearance and increased ROS levels contributed to the disease progression. Additionally, we showed that PLEKHM2-WT overexpression restored autophagic flux and ameliorated mitochondrial function, ultimately rescued PLEKHM2-deficient cardiomyopathy.

## Results

### Generation of homozygous PLEKHM2-KO hiPSCs and differentiating into cardiomyocytes

**Fig. 1:**
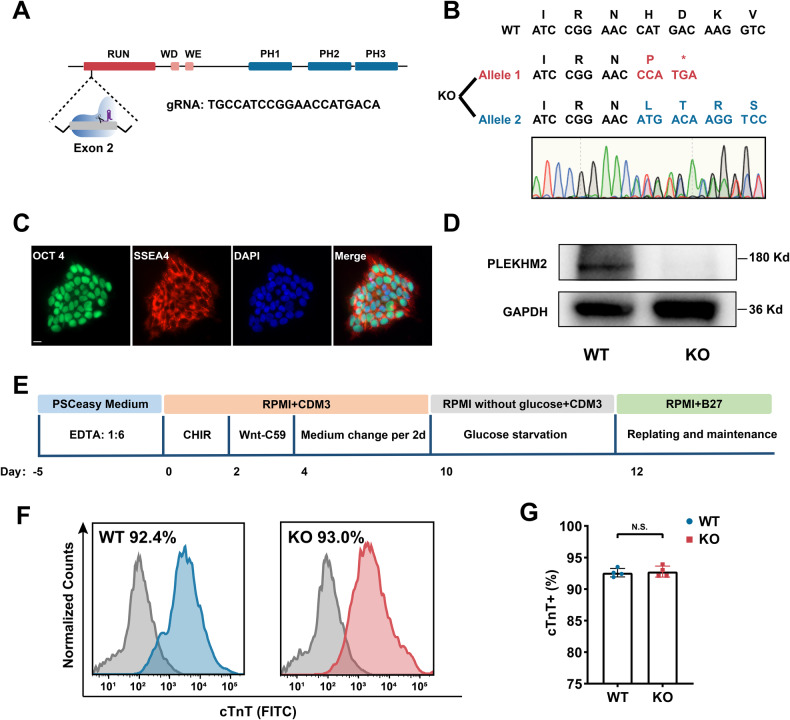
Generation of PLEKHM2-KO hiPSCs. **A** The *PLEKHM2* gene structure and the location of the guide RNA (gRNA) used for epigenome editing with CRISPR/Cas9. **B** Sequencing analysis confirmed a homozygous PLEKHM2-KO hiPSC line with a 1-nucleotide deletion in one allele and a 1-nucleotide insertion in the other allele. **C** Pluripotent stem cell markers SSEA4 and OCT4 were detected by immunofluorescence staining in PLEKHM2-KO colonies. Scale bar, 20 μm. **D** Western blot analysis of PLEKHM2 in WT hiPSC-CMs and PLEKHM2-KO hiPSC-CMs at day 20. **E** Protocol of small molecule-based methods to induce cardiac differentiation. **F**, **G** Flow cytometry analysis for cTnT from representative WT and PLEKHM2-KO differentiation at day 20. The results are presented as means ± SD of 3 independent experiments. N.S. not significant.

The generation of homozygous PLEKHM2-KO hiPSCs was carried out using the CRISPR-Cas9 system, and the guide RNA (gRNA) was designed and synthesized to target exon 2 of *PLEKHM2* (Fig. 1A). Subsequent screening confirmed the successful knockout of *PLEKHM2* gene, which revealed a one-nucleotide deletion in one allele and a one-nucleotide insertion in another allele (Fig. 1B and Supplementary Fig. 1B). The cell line under investigation was found to express the human pluripotency markers SSEA4 and OCT4 (Fig. 1C) and tested negative for mycoplasma contamination (Supplementary Fig. 1C). Western blot (WB) analyses revealed the absence of PLEKHM2 protein expression in PLEKHM2-KO hiPSC-CMs at day 20 (Fig. 1D). Wild-type (WT) and PLEKHM2-KO hiPSCs were differentiated into cardiomyocytes using the small molecule-based method (Fig. 1E). The efficacy of hiPSC-CMs differentiation was evaluated using flow cytometry, which indicated that PLEKHM2-KO and WT hiPSC-CMs exhibited similar proportions of cardiac Troponin T (cTnT)-positive cells (around 93%) at day 20 post differentiation (Fig. 1F, G). These results indicate that the PLEKHM2-KO hiPSC-CMs were successfully constructed.

### Impaired myocardial contractility and calcium handling in PLEKHM2-KO hiPSC-CMs

We next investigate the dynamic changes in myocardial contractility and calcium transients of PLEKHM2-KO hiPSC-CMs. The HCell series single myocardial cell function detection system was utilized to measure myocardial contractility [10] (Supplementary Fig. 2A), and the green fluorescent calcium-modulated protein 6 fast type (GCaMP6f) calcium imaging system was employed to track myocardial calcium transients [11] (Supplementary Fig. 2B, C).

**Fig. 2:**
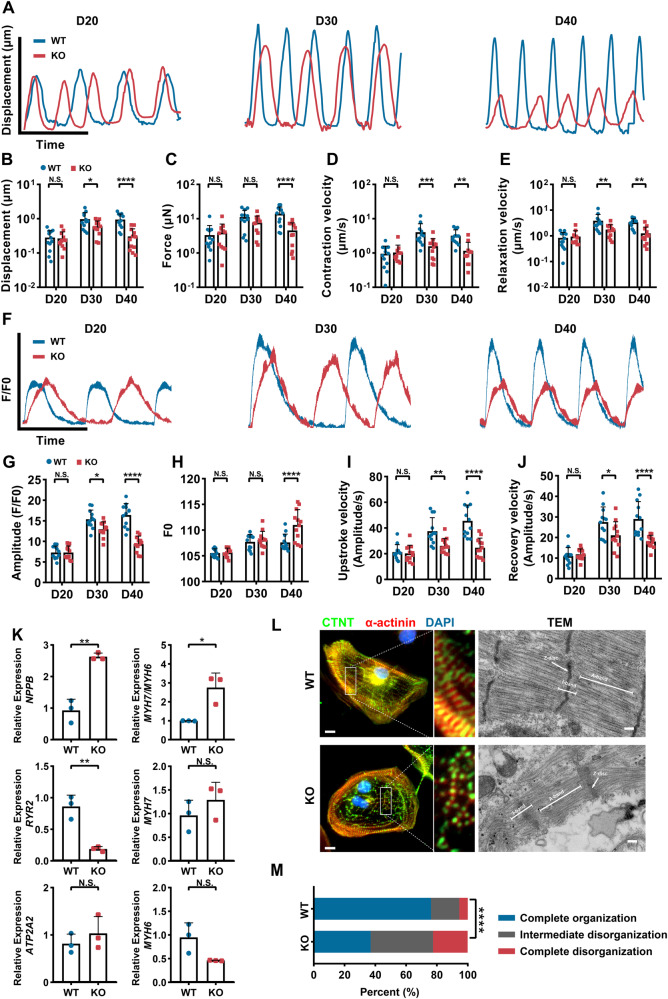
PLEKHM2-KO hiPSC-CMs develop impaired myocardial contractility and abnormal calcium handling. **A** Representative line scan images of myocardial contractility in WT hiPSC-CMs and PLEKHM2-KO hiPSC-CMs at days 20, 30, and 40. **B**–**E** Quantification of displacement, force, contraction and relaxation velocity in WT hiPSC-CMs and PLEKHM2-KO hiPSC-CMs (*n* = 12 cells per group). **F** Representative line scan images of calcium transients in WT hiPSC-CMs and PLEKHM2-KO hiPSC-CMs at days 20, 30, and 40. **G**–**J** Quantification of amplitude, diastolic Ca^2+^ concentration, upstroke and recovery velocity in WT hiPSC-CMs and PLEKHM2-KO hiPSC-CMs (*n* = 12 cells per group). **K** Quantitative PCR analysis of heart failure and calcium handling -related genes in WT hiPSC-CMs and PLEKHM2-KO hiPSC-CMs at days 40. Data are shown as mean ± SD of 3 independent experiments. **L** Representative immunofluorescence staining and transmission electron microscope (TEM) of sarcomeric. **M** Quantification of complete organization, intermediate disorganization, and complete organization in WT hiPSC-CMs and PLEKHM2-KO hiPSC-CMs at days 40 based to immunofluorescence staining (more than 120 cells per group). Scale bar, 10 μm. *p < 0.05; **p < 0.01; ***p < 0.001; ****p < 0.0001; N.S. not significant.

At the early stage of myocardial differentiation, specifically on 20 day, no significant alterations in myocardial contractility were observed between the WT hiPSC-CMs and the PLEKHM2-KO hiPSC-CMs. But at day 30, PLEKHM2-KO hiPSC-CMs exhibited a minor reduction in systolic displacement, as well as systolic and diastolic velocities compared to WT hiPSC-CMs, but no change in contractile force. And at day 40, the systolic displacement, contractile force, as well as systolic and diastolic velocities were significantly reduced in PLEKHM2-KO hiPSC-CMs compared to the WT hiPSC-CMs (Fig. 2A–E, and Supplementary Fig. 2D, E), showing that the PLEKHM2-KO hiPSC-CMs developed systolic dysfunction phenotype. Calcium transient is a principal mechanism responsible for myocardial contraction, wherein the magnitude of contraction force is contingent upon variations in calcium ion concentration within the cell [12]. Hence, we next evaluated the alterations in calcium transients in the myocardium. In accordance with the trend observed in myocardial contractility, no significant variation was detected in calcium transient of PLEKHM2-KO hiPSC-CMs during the early phase post myocardial differentiation. However, a decline in calcium transient amplitude was observed at day 30, alongside a decrease in upstroke and recovery velocity of calcium transients in PLEKHM2-KO hiPSC-CMs compared to WT hiPSC-CMs, and further exacerbated by 40 day (Fig. 2F–J, and Supplementary Fig. 2F). Interestingly, we also found that compared to WT hiPSC-CMs, the baseline values of calcium transients in PLEKHM2-KO hiPSC-CMs showed a significantly increased at day 40, indicating abnormal calcium handling in PLEKHM2-KO hiPSC-CMs (Supplementary Fig. 2G). These results suggest that abnormal calcium handling is a potential cause of the impaired myocardial contractility in PLEKHM2-deficient cardiomyopathy.

Subsequently, we evaluated the expression of key genes involved in heart failure and calcium handling. We found a significant increase in the expression of both *NPPB* and the *MYH7/MYH6* ratio in PLEKHM2-KO hiPSC-CMs compared to WT hiPSC-CMs, whereas ryanodine receptor 2 (*RYR2*) expression significantly decreased at day 40 (Fig. 2K). Moreover, we observed significantly disordered sarcomeres in PLEKHM2-KO hiPSC-CMs at day 40 (Fig. 2L, M). Moreover, transmission electron microscopy (TEM) showed significant abnormalities in the myofilaments of PLEKHM2-KO hiPSC CMs, including disordered myofilament arrangement and blurred Z-disc morphology (Fig. 2L). Overall, these findings corroborate the strong link between PLEKHM2 deficiency and DCM, which manifests as reduced contractility and impaired calcium handling, along with sarcomeric disorganization and dysregulated expression of heart failure markers.

### Distinct abnormalities genes expression in PLEKHM2-KO hiPSC-CMs

**Fig. 3:**
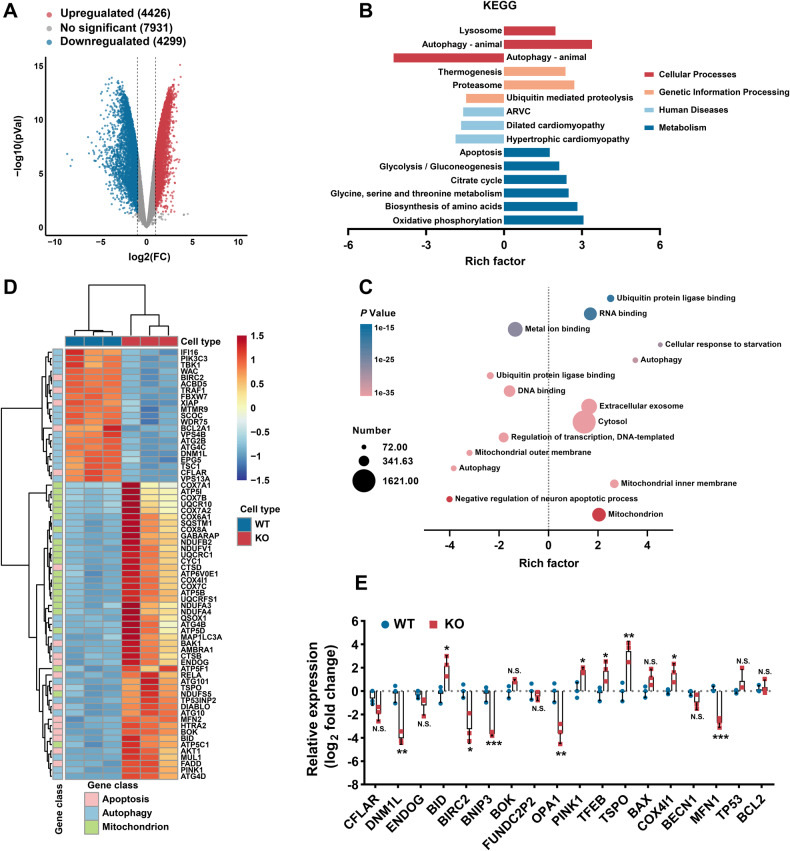
PLEKHM2 deficiency leads to abnormal expression of autophagy and mitochondria-related pathways. **A** Volcano plot shows 8725 genes with altered expression in PLEKHM2-KO hiPSC-CMs compared with WT. Blue and red dots indicate genes with increased and decreased expression, respectively, based on a *P* value < 0.05 and a log_2_ fold change >1 (*n* = 3 for each group). **B** Enrichment analysis using Kyoto Encyclopedia of Genes and Genomes (KEGG) databases revealed that pathways related to lysosomal function, autophagy, cardiomyopathy, apoptosis and metabolism were disrupted in PLEKHM2-KO hiPSC-CMs. ARVC, arrhythmogenic right ventricular cardiomyopathy. **C** Gene Ontology (GO) enrichment analysis showed significant changes in gene expression associated with mitochondrial function, apoptosis and autophagy in PLEKHM2-KO hiPSC-CMs. The color scale indicates the *P* values of the top 15 altered pathways in GO molecular function and the bubble size reflects the number of genes involved in each pathway. **D** Heatmap of differentially expressed genes involved in mitochondrial function, apoptosis and autophagy. **E** Quantitative PCR confirmed the altered expression of a representative subset of genes identified by RNA sequencing in PLEKHM2-KO hiPSC-CMs. Data are shown as mean ± SD of three independent experiments. *p < 0.05; **p < 0.01; ***p < 0.001; ****p < 0.0001; N.S., not significant.

To assess for potentially pathogenic effects of PLEKHM2-deficient cardiomyopathy, we performed quantitative transcriptome profiling by RNA-seq (Supplementary Fig. 3A–C). We identified 8725 differentially expressed genes in PLEKHM2-KO hiPSC-CMs versus WT hiPSC-CMs at day 40, including 4426 upregulated and 4299 downregulated genes (Fig. 3A). Kyoto Encyclopedia of Genes and Genomes (KEGG) analysis suggested that these dysregulated genes were enriched in pathways mainly involved in regulating autophagy, lysosome, cardiomyopathy, apoptosis and metabolism (Fig. 3B). Of particular a significant finding was that the dysregulated genes were enriched in autophagy with the highest enrichment score in PLEKHM2-KO hiPSC-CMs compared to WT hiPSC-CMs (Fig. 3B). The molecular-level analysis of Gene Ontology (GO) enrichment demonstrated a marked dysregulation in gene expression related to mitochondria, apoptosis, and autophagy in PLEKHM2-KO hiPSC-CMs (Fig. 3C, D). Notably, mitochondria-related pathways show the most significant differences between PLEKHM2-KO and WT hiPSC-CMs (Fig. 3C). Overall, these results indicate that PLEKHM2 deficiency leads to widespread dysregulation of signaling pathways in cardiomyocytes. Subsequently, we conducted quantitative PCR to validate the expression of genes associated with substantial dysregulation of mitochondria, apoptosis, and autophagy in RNA seq. Our results indicate a significant downregulation of *BNIP3*, *DNM1L*, *OPA1*, and *MFN1* in addition to an upregulation of *TSPO* expression in PLEKHM2-KO hiPSC-CMs compared to WT hiPSC-CMs at day 40 (Fig. 3E). It is widely acknowledged that BNIP3 and TSPO participate in various physiological processes such as mitophagy, apoptosis, oxidative stress, and the oxidative respiratory chain [13, 14]. While DNM1L, OPA1, and MFN1 play crucial roles in maintaining and regulating mitochondrial morphology and stability [15, 16]. The observed dysregulation in these genes highlight a disruption of mitochondrial homeostasis in PLEKHM2-deficient cardiomyopathy.

### Abnormal mitochondrial morphology and impaired mitochondrial clearance in PLEKHM2-KO hiPSC-CMs

**Fig. 4:**
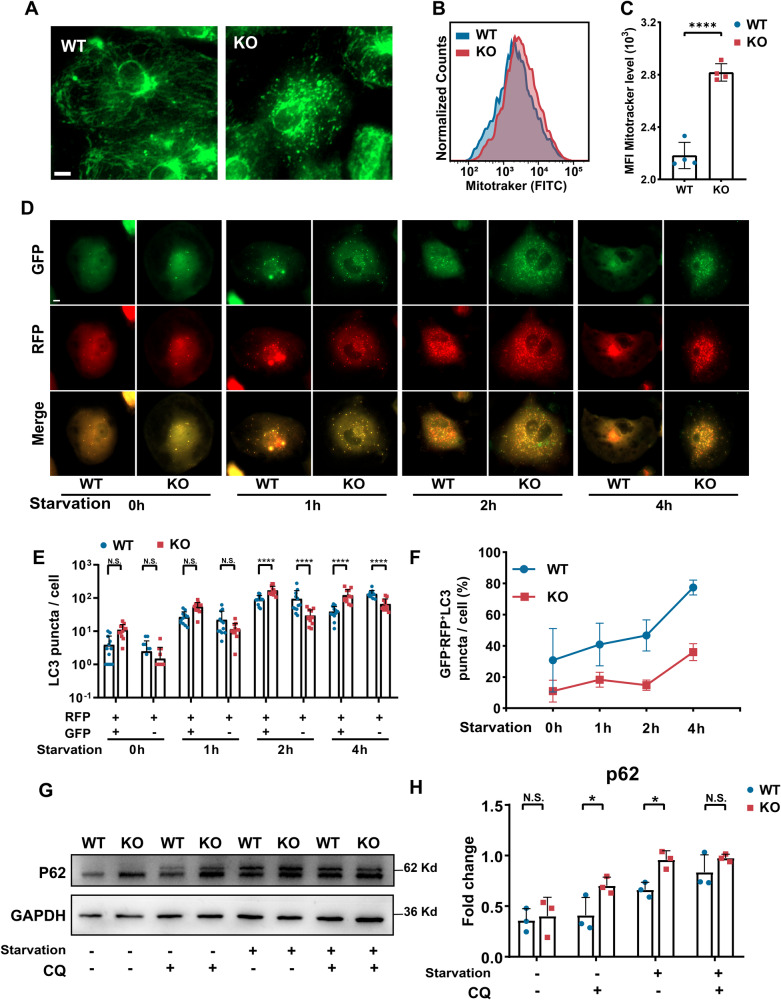
PLEKHM2 deficiency causes impaired autophagy and mitochondrial clearance. **A** Mitotracker staining revealed that PLEKHM2-KO altered the mitochondrial structure from the filamentous form aligned with the sarcomere in WT hiPSC-CMs to a punctate and fragmented morphology at day 40. Scale bar, 10 μm. **B**, **C** Quantification of Mitotracker green intensity obtained by flow cytometry demonstrates a significantly increased fluorescence intensity in PLEKHM2-KO hiPSC-CMs at day 40 as compared with WT hiPSC-CMs (*n* = 4). **D**–**F** Autophagic flux was assessed in WT hiPSC-CMs and PLEKHM2-KO hiPSC-CMs using mRFP-EGFP-LC3 adenovirus and subjected them to starvation medium for 0, 1, 2, and 4 h at day 40. Representative images and quantification of GFP+, RFP+, and GFP−, RFP+ puncta are shown in (**D**)–(**F**). 12 cells per cell line per condition were analyzed. Scale bars, 10 μm. **G**, **H** Representative western blot and quantification of P62 expression in WT hiPSC-CMs and PLEKHM2-KO hiPSC-CMs at day 40 (*n* = 3). CQ: chloroquine. Data are shown as mean ± SD. *p < 0.05; **p < 0.01; ***p < 0.001; ****p < 0.0001; N.S. not significant.

RNA-seq analysis revealed notable anomalies in autophagy and mitochondrial-related pathways, as indicated by significant findings in the KEGG and GO enrichment analysis. Following this discovery, we proceeded to investigate alterations in mitochondrion and autophagic processes. To further investigate the impacts of PLEKHM2 deficiency on the mitochondrion, we next assessed mitochondrial morphology and content using Mitotracker at day 40. Compared to the typical linear arrangement of mitochondria along sarcomeres in WT hiPSC-CMs, the mitochondria within PLEKHM2-KO hiPSC-CMs display distinctive fragmented and punctate patterns, along with irregular distribution throughout the cytoplasm (Fig. 4A, and Supplementary Fig. 4A, B). TEM revealed matrix swelling, empty spaces, and loose, disordered, and wider cristae in PLEKHM2-KO hiPSC-CMs (Supplementary Fig. 4A, C). This suggest that the PLEKHM2 deficiency significantly affects the localization and tissue structure of mitochondria in cardiomyocytes. Mitochondrial morphology disruption usually trigger mitophagy, which targets damaged or dysfunctional mitochondria for degradation and clearance from the cell, and the number of mitochondria within the cell usually decrease due to their removal [17]. However, further analysis using flow cytometry revealed an increasing mitochondrial content within the PLEKHM2-KO hiPSC-CMs, compared to the WT hiPSC-CMs (Fig. 4B, C). These results suggested that mitochondrial morphological abnormalities and impaired mitochondrial clearance occur in PLEKHM2-KO hiPSC-CMs.

The damaged mitochondria undergo degradation and digestion with the participation of lysosomes [4], while impaired autophagy or lysosomal acidification disorders usually impair mitophagy, resulting in delayed clearance of damaged mitochondria [18]. Consequently, we proceeded to investigate alterations in lysosomal localization and autophagy in the PLEKHM2-KO hiPSC-CMs. In this study, we utilized LAMP1 as a marker to identify lysosomal localization. Our results indicate that lysosomes in PLEKHM2-KO hiPSC-CMs exhibit significant clustering around the nucleus, whereas the lysosomes in the WT hiPSC-CMs demonstrate scattered distribution throughout the cytoplasm (Supplementary Fig. 4C, D), which is consistent with previous study [2]. To investigate the effects of PLEKHM2 deficiency on autophagy, we next monitored alterations in autophagic flux at 0, 1, 2, and 4 hours post-starvation. Day 3 after Ad-mRFP-EGFP-LC3 infection, we observed a significant accumulation of autophagosomes (GFP^+^/RFP^+^ puncta) with increasing starvation duration in both WT hiPSC-CMs and PLEKHM2-KO hiPSC-CMs. Notably, at the 2-hour after starvation, the number and proportion of autophagosome-lysosome fusion (GFP^-^/RFP^+^ puncta) in PLEKHM2-KO hiPSC-CMs was significantly lower than that of the WT hiPSC-CMs, indicating that the autophagic degradation of PLEKHM2-KO hiPSC-CMs was impaired (Fig. 4D–F). In this study, we observed that in the late phase of autophagy (4 hour after staving), most autophagosomes in the WT group accumulated around the nucleus and fused with lysosomes to form autolysosomes [19]. However, in the PLEKHM2-KO hiPSC-CMs, a substantial number of autophagosomes remained scattered within the cytoplasm and were not yet concentrated around the nucleus (Supplementary Fig. 4E), suggesting that PLEKHM2 deficiency affected the aggregation of autophagsomes to the perinuclear and fusion with lysosomes. In the subsequent WB results, we also observed that PLEKHM2 deficiency led to accumulation of p62 (Fig. 4G, H, and Supplementary Fig. 4F, G). In summary, these findings suggested that PLEKHM2 deficiency lead to abnormal lysosomal localization and blocking of autophagic flux, resulting in impaired autophagy and damaged mitochondrial accumulation.

### PLEKHM2 deficiency caused extensive mitochondrial dysfunction and excessive oxidative stress

**Fig. 5:**
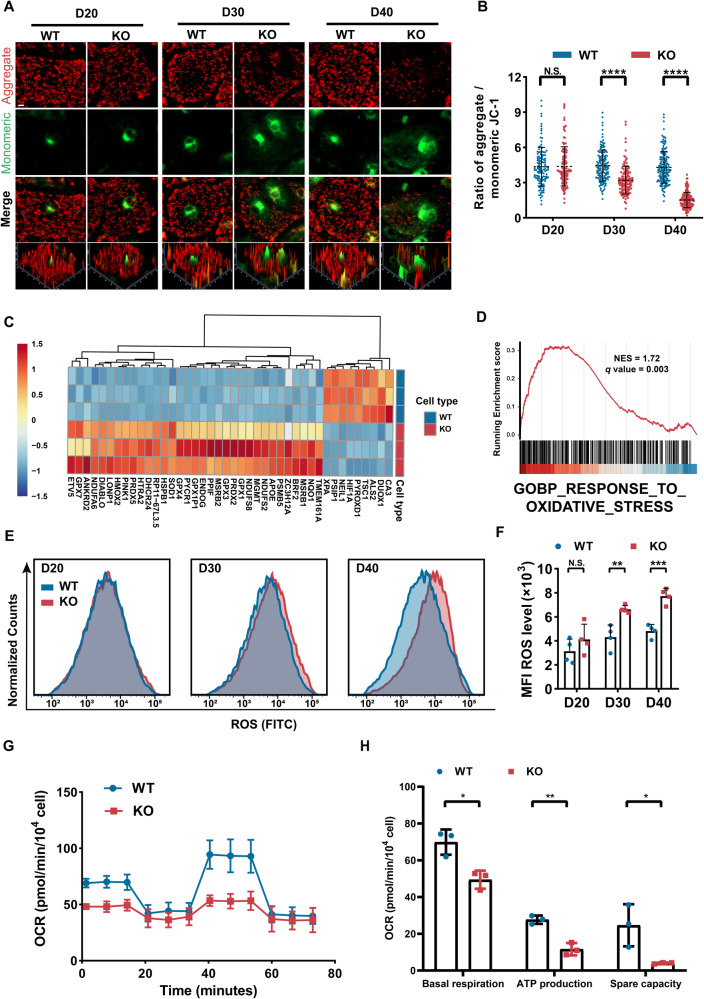
PLEKHM2 deficiency impairs mitochondrial function and induces ROS generation. **A**, **B** Representative immunofluorescence staining and quantification of *JC-1* revealed that mitochondrial monomers (green fluorescence) increased and the mitochondrial aggregates (red fluorescence) decreased gradually in PLEKHM2-KO hiPSC-CMs compared to WT at day 20, 30, and 40 (more than 120 cells per group). Scale bar, 10 μm. **C** Heatmap of differentially expressed genes involved in oxidative stress in PLEKHM2-KO hiPSC-CMs compared to WT hiPSC-CMs. **D** GSEA analysis revealed dysregulation of the respose to oxidative stress signaling pathway in PLEKHM2-KO hiPSC-CMs. **E**, **F** Representative flow cytometry analysis and quantification of cell reactive oxygen species (ROS) intensity demonstrated a continuous increased fluorescence intensity in PLEKHM2-KO hiPSC-CMs at days 20, 30, and 40 as compared with WT hiPSC-CMs. **G**, **H** Oxygen consumption rate (OCR) of WT hiPSC-CMs and PLEKHM2-KO hiPSC-CMs at 40 day was measured using a seahorse analyzer. Data are shown as mean ± SD. *p < 0.05; **p < 0.01; ***p < 0.001; ****p < 0.0001; N.S., not significant.

Mitophagy is a fundamental cellular self-cleaning mechanism that plays a critical role in maintaining mitochondrial function and preventing the accumulation of reactive oxygen species (ROS) by selectively removing damaged mitochondria [20, 21]. Δψm is a crucial indicator of mitochondrial health and function. To investigate the impact of PLEKHM2 deficiency on mitochondrial function, the carbocyanine compound *JC-1*, a fluorescent voltage-sensitive dye that possesses membrane-permeant fluorescent lipophilic cationic properties, was utilized to assess Δψm and mitochondrial health. Our results revealed that *JC-1* in PLEKHM2-KO hiPSC-CMs, exhibited a robust red fluorescence and weak green fluorescence similar to the WT hiPSC-CMs at day 20. However, over time, the red fluorescence of *JC-1* in the PLEKHM2-KO hiPSC-CMs decreased gradually, while the green fluorescence increased (Fig. 5A). Notably, at day 30 and 40, the ratio of aggregate to monomeric *JC-1* fluorescence in the PLEKHM2-KO hiPSC-CMs significantly reduced compared to that of the WT hiPSC-CMs (Fig. 5B). Futhrtmore, the destabilization in Δψm lead to the release of cytC from mitochondria, which activated the caspase-3 in PLEKHM2-KO hiPSC-CMs at 40 day (Supplementary Fig. 5A–C). These results suggest that PLEKHM2 deficiency leads to extensive depolarization of Δψm and impaired mitochondrial function.

Numerous studies have shown that ROS plays a crucial role in inducing widespread Δψm depolarization by directly triggering mPTP opening. And mPTP opening can further enhance ROS production by impairing Δψm, ultimately triggering a vicious cycle of Δψm depolarization and ROS production [22–24]. RNA-seq suggested significant changes in the expression gene profile associated with oxidative stress in PLEKHM2-KO hiPSC-CMs compared to WT hiPSC-CMs (Fig. 5C, D). Thus, to investigate whether the PLEKHM2 deficiency leads to an increase in ROS levels, we assessed the dynamic changes in ROS levels in PLEKHM2-KO hiPSC-CMs. We found a continuous increase in ROS levels in PLEKHM2-KO hiPSC-CMs than WT hiPSC-CMs (Fig. 5E, F), which indicated that PLEKHM2 deficiency causes progressive oxidative stress in hiPSC-CMs. To further investigate the effect of PLEKHM2 deficiency on mitochondrial OXPHOS activity, the oxygen consumption rates (OCR) of WT hiPSC-CMs and PLEKHM2-KO hiPSC-CMs was analyzed at day 40 (Fig. 5G). These kinetic results revealed that PLEKHM2 deficiency significantly impaired ATP production, basal respiration and spare capacity (Fig. 5H). These results suggest that PLEKHM2 deficiency causes extensive mitochondrial dysfunction.

### Oxidative stress exacerbates PLEKHM2-deficient cardiomyopathy

**Fig. 6:**
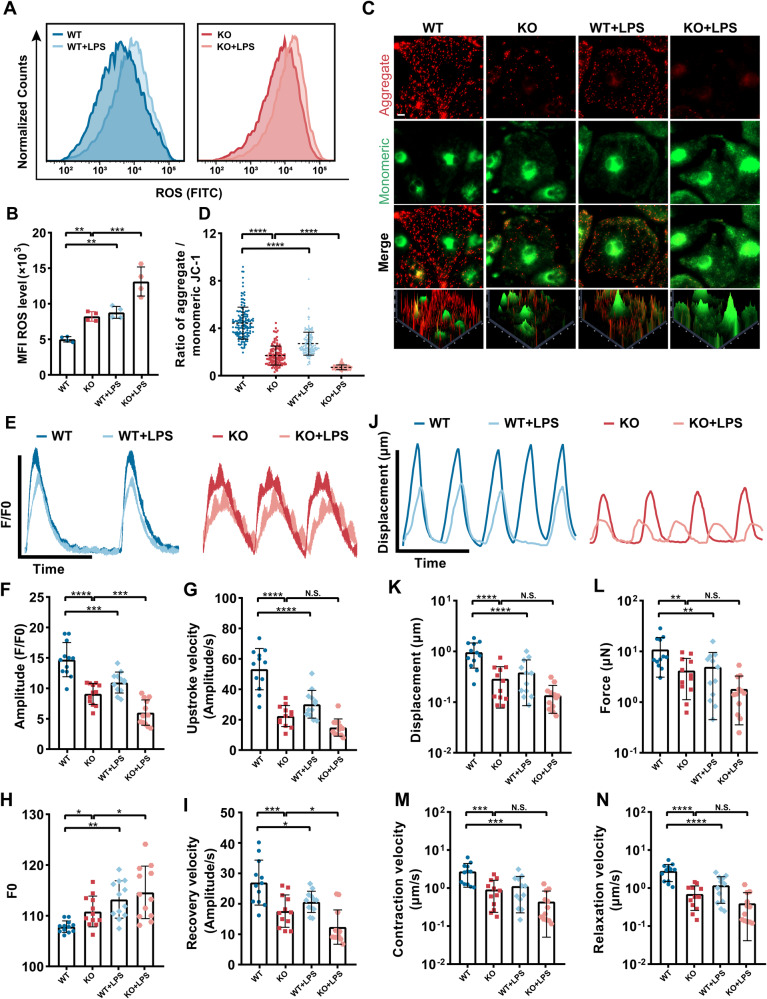
PLEKHM2 deficiency exacerbates LPS-induced mitochondrial dysfunction and impaired myocardial contractility. **A**, **B** Representative flow cytometry analysis and quantification of cellular ROS levels showed that both WT hiPSC-CMs and PLEKHM2-KO hiPSC-CMs had significantly increased ROS production compared to untreated controls after LPS exposure (*n* = 4 independent experiments). **C**, **D** Representative immunofluorescence staining and quantification of *JC-1* revealed that LPS treatment impaired mitochondrial membrane potential of WT hiPSC-CMs and PLEKHM2-KO hiPSC-CMs, as indicated by increased green fluorescence (monomeric form) and decreased red fluorescence (aggregated form) of *JC-1* (more than 120 cells per group). Scale bar, 10 μm. **E** Representative line scan images of calcium transients in WT hiPSC-CMs and PLEKHM2-KO hiPSC-CMs with or without LPS treatment at day 40. **F**–**I** Quantification of amplitude, diastolic Ca^2+^ concentration, upstroke and recovery velocity in WT hiPSC-CMs and PLEKHM2-KO hiPSC-CMs (*n* = 12 cells per group) with or without LPS treatment at day 40. **J** Representative line scan images of myocardial contractility in WT hiPSC-CMs and PLEKHM2-KO hiPSC-CMs with or without LPS treatment at day 40. **K**–**N** Quantification of displacement, force, contraction and relaxation velocity in WT hiPSC-CMs and PLEKHM2-KO hiPSC-CMs with or without LPS treatment at day 40 (*n* = 12 cells per group). Data are shown as mean ± SD. *p < 0.05; **p < 0.01; ***p < 0.001; ****p < 0.0001; N.S., not significant.

Previous studies have shown a strong link between oxidative stress and cardiomyopathy. To investigate whether ROS plays an important role in the pathogenesis of PLEKHM2-deficient cardiomyopathy, we administered oxidative stress activator, lipopolysaccharides (LPS) to WT hiPSC-CMs and PLEKHM2-KO hiPSC-CMs, and observed the effects on myocardial mitochondrial function, calcium handling, and contractility at day 40. After LPS administration, both WT hiPSC-CMs and PLEKHM2-KO hiPSC-CMs exhibited significantly higher levels of ROS than untreated CMs (Fig. 6A, B). Next, the *JC-1* was utilized to assess the effect of LPS treatment on mitochondrial function. Our results showed that LPS treatment induced the same mitochondrial dysfunction phenotype in WT hiPSC-CMs as in PLEKHM2-KO hiPSC-CMs. Moreover, LPS treatment exacerbated the Δψm destabilization in PLEKHM2-KO hiPSC-CMs compared to untreated hiPSC-CMs (Fig. 6C, D). To investigate whether oxidative stress accelerates the progression of PLEKHM2-deficient cardiomyopathy, we evaluated the effects of LPS administration on the calcium transient and myocardial contractility of WT hiPSC-CMs and PLEKHM2-KO hiPSC-CMs. We found that LPS treatment decreased calcium transients (Fig. 6E–I, and Supplementary Fig. 6) in both WT hiPSC-CMs and PLEKHM2-KO hiPSC-CMs compared to untreated hiPSC-CMs. And LPS treatment significantly decreased myocardial contractility (Fig. 6J–N) in WT hiPSC-CMs compared to untreated hiPSC-CMs. These results suggested that oxidative stress may play a significant role in mitochondrial dysfunction, abnormal calcium handling and impaired myocardial contractility in the development of PLEKHM2-deficient cardiomyopathy.

### Inhibiting oxidative stress partially rescued PLEKHM2-deficient cardiomyopathy

**Fig. 7:**
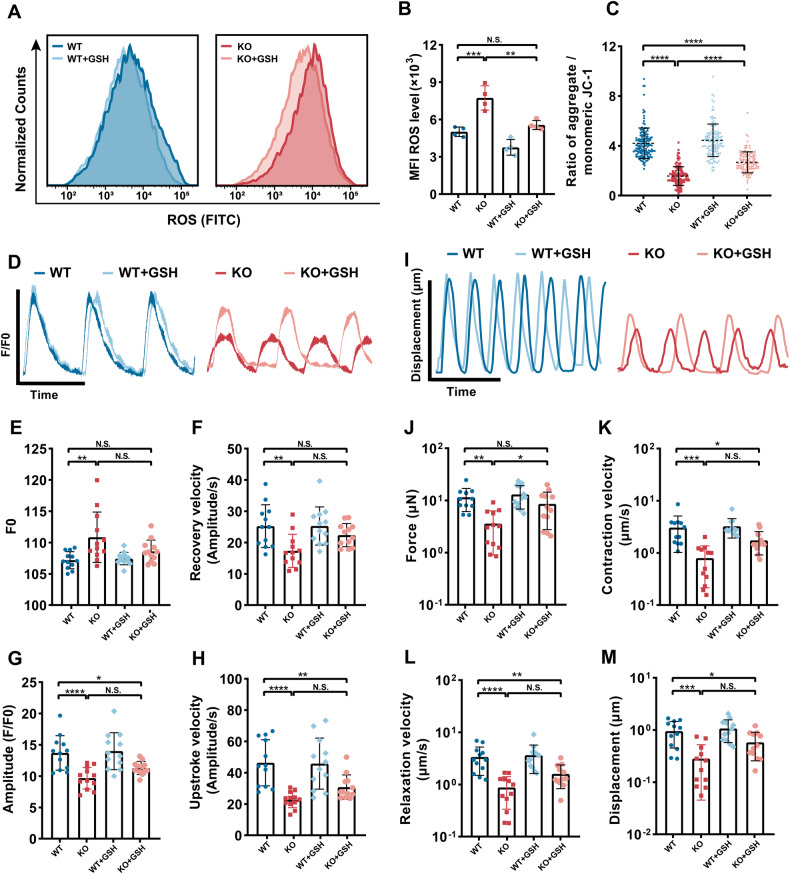
Inhibiting ROS rescues PLEKHM2 deficiency-induced disease phenotypes. **A**, **B** Representative flow cytometry analysis and quantification of cellular ROS levels showed that underwent GSH treatment PLEKHM2-KO hiPSC-CMs exhibited a considerable reduction in ROS levels comparable to WT hiPSC-CMs (*n* = 4 independent experiments). **C** Quantification of *JC-1* revealed that GSH treatment increased significantly the Δψm level in PLEKHM2-KO hiPSC-CMs (more than 120 cells per group). **D** Representative line scan images of calcium transients of WT hiPSC-CMs and PLEKHM2-KO hiPSC-CMs with or without GSH treatment at day 40. **E**–**H** Quantification of amplitude, diastolic Ca^2+^ concentration, upstroke and recovery velocity in WT hiPSC-CMs and PLEKHM2-KO hiPSC-CMs with or without GSH treatment at day 40 (*n* = 12 cells per group). **I** Representative line scan images of myocardial contractility in WT hiPSC-CMs and PLEKHM2-KO hiPSC-CMs with or without GSH treatment at day 40. **J**–**M** Quantification of displacement, force, contraction and relaxation velocity in WT hiPSC-CMs and PLEKHM2-KO hiPSC-CMs with or without GSH treatment at day 40 (*n* = 12 cells per group). Data are shown as mean ± SD. *p < 0.05; **p < 0.01; ***p < 0.001; ****p < 0.0001; N.S., not significant.

Reduced glutathione (GSH) is an important antioxidant helps to prevent and reduce oxidative stress by neutralizing free radicals, widely used in the treatment of various types of oxidative stress-related diseases, including neurodegenerative diseases, cardiovascular diseases, and diabetes [25]. Hence, we treated PLEKHM2-KO hiPSC-CMs with GSH at day 30 to observe whether it could rescue the disease phenotype caused by PLEKHM2 deficiency. We found that PLEKHM2-KO hiPSC-CMs treated with GSH exhibited a considerable reduction in ROS levels (Fig. 7A, B) and significantly elevation of Δψm level compared to untreated PLEKHM2-KO hiPSC-CMs (Fig. 7C, and Supplementary Fig. 7A). This indicates that inhibiting ROS helps improve mitochondrial function by preventing oxidative stress-induced damage to nearby mitochondria. Next, we observed the effects of GSH on the calcium transient and myocardial contractility of PLEKHM2-KO hiPSC-CMs. After GSH treatment, the diastolic calcium concentration and recovery velocity of PLEKHM2-KO hiPSC-CMs were comparable to that of WT hiPSC-CMs (Fig. 7D-H, and Supplementary Fig. 7B). Similarly, after GSH treatment, the PLEKHM2-KO hiPSC-CMs also showed significant improvements in contractile force (Fig. 7I–M). These results further suggested the critical role of oxidative stress in mediating the disease phenotype of PLEKHM2-deficient cardiomyopathy.

### PLEKHM2-WT overexpression restored autophagic flux and ameliorated PLEKHM2-deficient cardiomyopathy

Previous studies have shown that PLEKHM2 deficiency lead to abnormal lysosomal localization and impaired autophagic flux [2], causing damaged mitochondrial accumulation and ROS production. mTORC1 signaling pathway is the main negative regulator of autophagy, inhibiting autophagy by phosphorylating and inactivating key autophagy proteins such as ULK1 and ATG13 [26]. Hence, we investigated whether inhibition of mTORC1 by RAPA could boost autophagy and rescue the mitochondrial dysfunction and ROS generation in PLEKHM2-KO hiPSC-CMs. We found that p-mTOR levels were significantly higher in PLEKHM2-KO hiPSC-CMs than WT hiPSC-CMs (Supplementary Fig. 8A, B). Administration of RAPA significantly reduced the p-mTOR levels in PLEKHM2-KO hiPSC-CMs (Supplementary Fig. 8A, B). We further observed the effect of RAPA on autophagic flux in PLEKHM2-KO hiPSC-CMs. RAPA increased the number of autophagosomes (GFP^+^/RFP^+^ puncta) and autophagolysosome (GFP^-^/RFP^+^ puncta) in PLEKHM2-KO hiPSC-CMs, indicating that RAPA induced autophagy (Supplementary Fig. 8C). However, the number and proportion of GFP^-^/RFP^+^ puncta in PLEKHM2-KO hiPSC-CMs was still significantly lower than that WT hiPSC-CMs (Supplementary Fig. 8C), indicating that rapamycin cannot completely improve obstruction of autophagic flux caused by PLEKHM2 deficiency. We then evaluated the effects of RAPA treatment on mitochondrial function and myocardial contractility in PLEKHM2-KO hiPSC-CMs at day 40. We found that RAPA treatment partially improved Δψm level and reduced ROS generation in PLEKHM2-KO hiPSC-CMs (Supplementary Fig. 8E–G). Next, we observed the effects of RAPA treatment on myocardial contraction and calcium transient in PLEKHM2-KO hiPSC-CMs. We found that RAPA treatment enhanced the calcium transient amplitude (Supplementary Fig. 8H–L) of PLEKHM2-KO hiPSC-CMs, but the myocardial contractility (Supplementary Fig. 8M–R) was still significantly lower than those in WT hiPSC-CMs. These results indicated that administering RAPA cannot completely correct impaired autophagy caused by PLEKHM2 deficiency, but partially improves the disease phenotype of PLEKHM2-deficient cardiomyopathy.

**Fig. 8:**
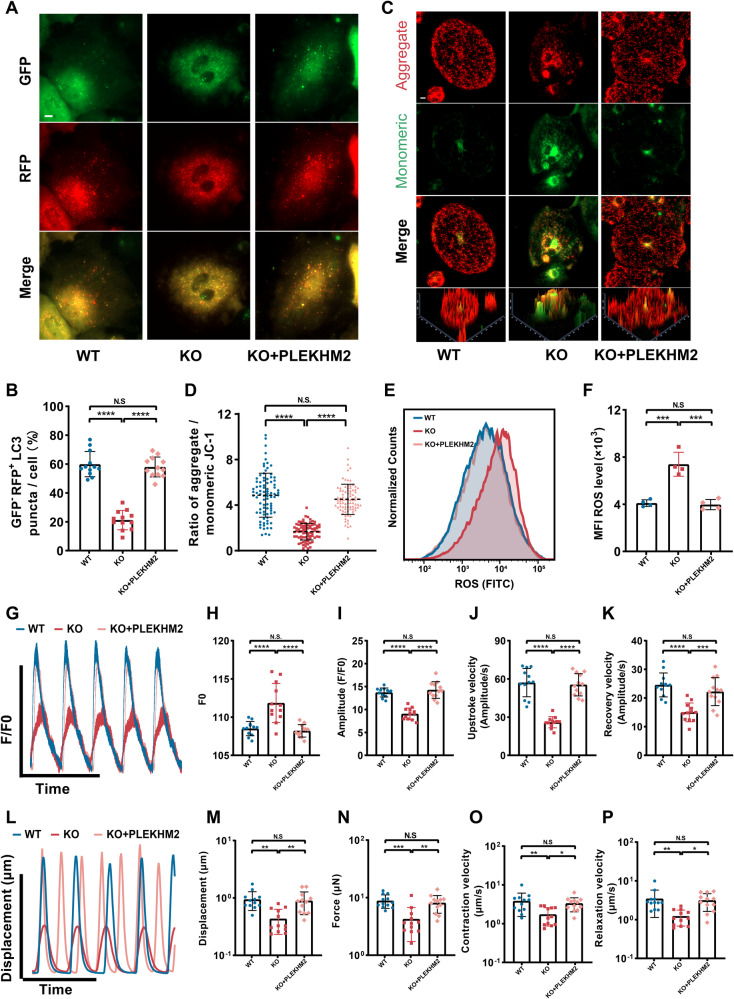
PLEKHM2-WT overexpression restored autophagic flux and ameliorated PLEKHM2-deficient cardiomyopathy. **A**, **B** Autophagic flux was assessed in hiPSC-CMs using mRFP-EGFP-LC3 adenovirus. Representative images and quantification of GFP^−^ and RFP^+^ puncta are shown in (**A**) and (**B**). **C**, **D** Representative immunofluorescence staining and and quantitative analysis of *JC-1* revealed that PLEKHM2-WT overexpression ameliorated Δψm of PLEKHM2-KO hiPSCs-CMs (more than 70 cells per group). Scale bars, 10 μm. **E**, **F** Representative flow cytometry analysis and quantification of cell reactive oxygen species (ROS) intensity demonstrated PLEKHM2-WT overexpression reduced ROS levels of PLEKHM2-KO hiPSCs-CMs. **G** Representative line scan images of calcium transients of WT hiPSC-CMs and PLEKHM2-KO hiPSC-CMs with or without PLEKHM2-WT overexpression at day 40. **H**–**K** Quantification of amplitude, diastolic Ca^2+^ concentration, upstroke and recovery velocity in WT hiPSC-CMs and PLEKHM2-KO hiPSC-CMs with or without PLEKHM2-WT overexpression at day 40 (*n* = 12 cells per group). **L** Representative line scan images of myocardial contractility in WT hiPSC-CMs and PLEKHM2-KO hiPSC-CMs with or without PLEKHM2-WT overexpression at day 40. **M**–**P** Quantification of displacement, force, contraction and relaxation velocity in WT hiPSC-CMs and PLEKHM2-KO hiPSC-CMs with or without PLEKHM2-WT overexpression at day 40 (*n* = 12 cells per group). Data are shown as mean ± SD. *p < 0.05; **p < 0.01; ***p < 0.001; ****p < 0.0001; N.S. not significant.

We next investigated whether PLEKHM2-WT overexpression could restore autophagic flux in PLEKHM2-KO hiPSC-CMs and rescued the disease phenotype of PLEKHM2-deficient cardiomyopathy. We found that PLEKHM2-WT overexpression corrected the abnormal lysosomal localization (Supplementary Fig. 9) and increased the number and proportion of GFP^-^/RFP^+^ puncta in PLEKHM2-KO hiPSC-CMs, compared to untreated PLEKHM2-KO hiPSC-CMs (Fig. 8A, B). This indicates that PLEKHM2-WT overexpression improve the autophagic degradation in PLEKHM2-KO hiPSC-CMs. We further observed the effects of PLEKHM2-WT overexpression on the mitochondrial function of PLEKHM2-KO hiPSC-CMs. PLEKHM2-KO hiPSC-CMs treated with PLEKHM2-WT overexpression exhibited a significant increase in the Δψm level and decrease in ROS levels compared to untreated PLEKHM2-KO hiPSC-CMs (Fig. 8C–F). Subsequently, we evaluated the effects of PLEKHM2-WT overexpression on the calcium transient and myocardial contractility of PLEKHM2-KO hiPSC-CMs. PLEKHM2-WT overexpression significantly enhanced calcium transient amplitude (Fig. 8G–K) and myocardial contractility (Fig. 8L–P) compared to untreated PLEKHM2-KO hiPSC-CMs. This further demonstrates that PLEKHM2 plays a crucial role in regulating autophagy and clearing damaged mitochondria.

## Discussion

Previous studies reported two cases of early onset DCM caused by *PLEKHM2* loss-of-function mutations, suggesting that *PLEKHM2* is a novel candidate gene for DCM [2, 3]. However, few studies have explored the deep molecular mechanisms of PLEKHM2 deficiency-mediated cardiomyopathy. Here, we established an in vitro model of PLEKHM2-KO hiPSC-CMs, and confirmed that PLEKHM2 deficiency caused impaired myocardial contractility and abnormal calcium handling. Subsequent RNA-seq showed an abnormal expression of pathways related to mitochondria, autophagy, and apoptosis in PLEKHM2-KO hiPSC-CMs. Importantly, our results suggested that impaired mitochondrial clearance with elevated ROS levels played an important role in the disease progression of PLEKHM2-deficient cardiomyopathy. And PLEKHM2-WT overexpression restored autophagic flux and ameliorated mitochondrial function, ultimately rescued PLEKHM2-deficient cardiomyopathy.

Lysosomal bidirectional transport is maintained by the network coordination and competition of some small GTPases, such as RAB7-RILP and ARL8-PLEKHM2 [5]. However, when the balance is disrupted by mutations, overexpression or inhibition of these small GTPases or their effectors, the spatial distribution of lysosomes becomes abnormal, causing lysosomal dysfunction and impared autophagy [27]. Prior studies have suggested that deletion or mutation of ARL8 not only leads to abnormal lysosomal localization, but also affects autophagosome-lysosome fusion by impairing its interaction with HOPS complex VPS-41, leading to the accumulation of autophagies [28]. In this study, we also found that PLEKHM2 deficiency leads to abnormal lysosomal localization, impaired autophagy and accumulation of autophagosome (Fig. 4). Interestingly, we also observed that in the late stages of autophagy, a substantial number of autophagysome remained scattered within the cytoplasm and failed to fuse with lysosomes in PLEKHM2-KO hiPSC-CMs, whereas most of the autophagysome in WT hiPSC-CMs had accumulated around the nucleus and fused with lysosomes to complete the autophagic degradation process. A possible explanation is that PLEKHM2 deficiency may affected the transport of autophagysome and autophagosome-lysosome fusion. RAB7 is a key regulator for perinuclear transport of autophagosomes and late endosomes [29]. And PLEKHM2 can negatively regulates RAB7 by recruiting TBC1D15, to catalyze its GTP hydrolysis and detachment from lysosomes, thereby maintaining the proper segregation of intralysosomal organelles and the redistribution of RAB7 in the cytoplasm [30]. However, PLEKHM2 deficiency may affect the inactivation of RAB7, which leads to abnormal distribution of RAB7 [2], ultimately resulting in delayed autophagosome transport to the perinuclear region and impaired autophagosome-lysosome fusion.

To maintain efficient contractile function, cardiomyocytes contain a high mitochondrial density to support their high metabolic demands [31]. However, mitochondria are susceptible to damage caused by various factors, including viruses, medications and severe oxidative stress. Hence, timely removal of damaged mitochondria by mitophagy helps myocardial cells to maintain normal mitochondrial function and prevent oxidative stress-mediated cellular damage [32]. When mitophagy is impaired, damaged mitochondria accumulate and release ROS, which can damage nearby healthy mitochondria and ultimately cause more mitochondrial dysfunction [33]. Our study also demonstrated that PLEKHM2-KO hiPSC-CMs accumulated a large number of morphologically and structurally defective mitochondria, accompanied by increased ROS levels and extensive Δψm destabilization. As a highly oxidative molecule, excessive ROS can damage the mitochondrial membrane structure and oxidize the mPTP, leading to Δψm destabilization of nearby healthy mitochondria [34]. Whereas Δψm destabilization in turn enhance ROS production [23]. This is possible cause of excessive ROS production and extensive Δψm destabilization in PLEKHM2-KO hiPSC-CMs.

Calcium transient is considered to be an important mechanism responsible for excitation-contraction coupling, and abnormal calcium handling often leads to impaired myocardial contractility [35]. In this study, we found reduced calcium transient amplitude and increased cytoplasmic calcium in PLEKHM2-KO hiPSC-CMs. Decreased calcium transient amplitude is an important factor causing systolic dysfunction in DCM [36], while increased cytoplasmic calcium often leads to severe ventricular arrhythmia [37]. This is consistent with the disease phenotype previously reported in cases of homozygous frameshift mutations in PLEKHM2, which were characterized by systolic dysfunction and ventricular arrhythmia [2]. Here, abnormal calcium transients in PLEKHM2-KO hiPSC-CMs may be due to multiple mechanisms, including excessive ROS production and extensive Δψm destabilization. The Δψm is an important factor for maintaining mitochondrial function and homeostasis, extensive destabilization of which can lead to abnormal release of calcium ion and apoptosis-inducing factor Cyt c, ultimately causing an increase in cytoplasmic calcium ions and irreversible cardiomyocytes loss [38]. Additionally, excessive ROS were considered to be able to affect the interaction of SR proteins by inducing post-translational modifications [39]. For instance, ROS can directly oxidize RyR2 and nitrosylate SERCA2a, or indirectly activate CAMKII to phosphorylate RYR2, leading to increased spontaneous calcium spark frequency and SR calcium leakage, as well as impaired SR calcium uptake [40–42].

Oxidative stress mediated by elevated ROS has been shown to be involved in the pathological mechanisms of various diseases, such as Barth syndrome [42], neurodegenerative diseases [43], atherosclerosis [44], and Duchenne cardiomyopathy [45] etc. Inhibition of ROS by administration of antioxidant drugs, such as GSH, glutathione peroxidase (GPX) mimicsa and superoxide dismutase (SOD) mimics, has been shown to slow disease progression and improve disease phenotype [42, 45, 46]. In PLEKHM2-deficient cardiomyopathy, ROS also played a critical role in mediating extensive Δψm destabilization and abnormal calcium handling. And treatment with GSH partially improved the Δψm and calcium transients in PLEKHM2-KO hiPSC-CMs. Therefore, these results suggest that ROS inhibition is a potential therapeutic strategy to rescue the disease phenotype of PLEKHM2-deficient cardiomyopathy.

In this study, we found that PLEKHM2 deficiency leads to impaired autophagy and a significant reduction in the number and ratio of autophagic lysosomes, resulting in impaired mitochondrial clearance. Numerous studies have shown that mTORC1 signaling pathway is a key negative regulator of autophagy [26], and inhibiting mTORC1 activation by RAPA can enhance autophagy and improve disease phenotype [47]. However, we found that RAPA treatment cannot completely correct impaired autophagy caused by PLEKHM2 deficiency, but partially improves the disease phenotype of PLEKHM2-deficient cardiomyopathy (Supplementary Fig. 8). Previous studies have indicated that PLEKHM2 not only mediates the anterograde transport of lysosomes, but also regulates the distribution of Rabs [2], which are essential for autophagy. Rab7 facilitates the transport of autophagosomes to the nuclear periphery [48], while Rab5 is involved in the formation of autophagy [49]. Thus, it is necessary to further elucidate the underlying mechanism of PLEKHM2 in regulating autophagy, which aid in the treatment of PLEKHM2-deficient cardiomyopathy.

Although hiPSCs have inherent limitations in mimicking the disease phenotypes of three-dimensional organs or tissues, our results suggested that PLEKHM2-KO hiPSC-CMs exhibits disease phenotypes such as impaired myocardial contractility and abnormal calcium handling. Moreover, our results revealed that impaired mitophagy and elevated ROS levels played important roles in the disease progression of PLEKHM2-deficient cardiomyopathy, and PLEKHM2-WT overexpression restored autophagic flux and improved mitochondrial function, ultimately rescued the disease phenotype of PLEKHM2 deficient cardiomyopathy.

## Materials and methods

### Generation of PLEKHM2 deficient hiPSC using CRISRP-Cas9 system

Wild-type (WT) iPSCs were obtained from the ZZUNEUi022-A cell line [50], which was previously established from healthy male urine cells. To generate PLEKHM2-KO hiPSCs, a single guide RNA (sgRNA) targeting exon 2 of *PLEKHM2* was designed by using the CRISPR design tool (http://crispr.mit.edu/). The epiCRISPR plasmid (Addgene, #135960) carrying gRNA was transfected into WT hiPSCs by electroporation. Clone selection using puromycin (0.5 μg/mL, Selleck Chemicals, Houston, TX, US) was performed on the third day after electrotransfer (Supplementary Fig. 1A). The surviving colonies were carefully chosen and transferred into a 48-well plate for further validation through Sanger sequencing (Sangon Biotech, Shanghai, China). This study was approved by the Ethics Review Committee of the First Affiliated Hospital of Zhengzhou University (2018-KY-38).

### Cell culture and myocardial differentiation

Matrigel (Corning Life Science, Tewksbury, MA, USA) coated 6-well plates were seeded with hiPSCs at a density of 1×10^5^ cells / well and cultured in PSCeasy medium (Cellapy, Beijing, China) at 37°C and 5% CO_2_. hiPSCs were differentiated into cardiomyocytes using the small molecule-based method as previously described [51]. Myocardial purification is performed by the lactate metabolism-selection method at day 10 post myocardial differentiation. And the cells were reseeded on new matrigel-coated culture plates. Here, the first day of myocardial differentiation was defined as Day 1.

### Quantitative PCR

Total mRNA was isolated from hiPSC-CMs using the TRIzol reagent (Thermo Fisher Scientific) and converted to cDNA using the PrimeScript™ RT Master Mix (Takara) according to the manufacture’s protocol. Then, We performed quantitative real-time PCR on a QuantStudio 3 instrument (Thermo Fisher Scientific) with TB Green Premix Ex Taq II (Takara) as the PCR mix. The relative quantification of target genes was calculated by the 2^−ΔΔCt^ method, using GAPDH as housekeeping gene to normalize gene expression. The primers sequences used are listed in Supplementary Table 1.

### Immunofluorescence staining

For immunofluorescence staining, purified hiPSC-CMs were seeded on cell climbing tablets and fixed with 4% paraformaldehyde for 10 min at room temperature. After washing with phosphate buffered saline (PBS), the cells were permeabilized with 0.2% Triton X-100 for 15 min and blocked with 4% bovine serum albumin (BSA) for 1 h at room temperature. The cells were then incubated with primary antibodies overnight at 4 °C. The next day, the cells were washed with PBS and incubated with corresponding secondary antibodies for 2 hour at room temperature, followed by DAPI (300 nM, Thermo Fisher Scientific) for 5 min. Images were acquired using an OLYMPUS IX73 microscope and analyzed by Zen software. The primary and secondary antibodies used and their dilutions are listed in Supplementary Table 2.

### Flow cytometry

Flow cytometry was used to assess the purity of hiPSC-CMs. The cells were digested and dissociated into single cells, then centrifuged at 1500 rpm and resuspended in 1 ml of 4% paraformaldehyde for 10 min at room temperature for fixation. After washing with PBS and centrifuging, the cells were resuspended in 1 ml of 4% BSA and incubated for 1 hour at room temperature to block non-specific antigens. Then, the cells were incubated with cTnT primary antibody (1:100) for 1 hour at room temperature, followed by washing with PBS and incubating with secondary antibody (1:100) for 30 min. After washing and resuspending with PBS, the cells were analyzed by flow cytometry for hiPSC-CMs purity, using IgG isotype control antibody as a reference for background staining. The primary and secondary antibodies used and their dilutions are listed in Supplementary Table 2.

To quantify mitochondria and ROS levels, hiPSC-CMs were digested and were incubated with 50 nM of MitoTracker (Beyotime, Shanghai, China) and 10 mM CellROX Green (Thermo Fisher Scientific) in medium for 20 min at 37 °C in a 5% CO2 incubator. The samples were measured with a BD FACS Canto II flow cytometer (BD Biosciences, US) and analyzed by using FlowJo X software.

### Myocardial contractility

Plexithermo HCell series single myocardial cell function detection system was used to measure the force of myocardial contractility [52]. Briefly, hiPSC-CMs were seeded on micropatterned hydrogel plates (Plexithermo, Singapore) that carried red fluorescent beads. The beads adhered to the hiPSC-CMs and allowed for tracking their displacement and velocity as they contracted under a fluorescence microscope. Videos of the hiPSC-CMs were recorded using an OLYMPUS IX73 microscope for 10 s and saved in uncompressed avi format. The video data was analyzed with the Hcell software (Plexithermo, Singapore) provided by the hydrogel system and the results were exported to excel files.

### Ca^2+^ imaging

To investigate the calcium handling, we generated WT-GCaMP and PLEKHM2-KO-GCaMP hiPSC as previously described [11]. Briefly, after electroporing AAVS1_sgRNA (Addgene, #100554) and pAAVS1-PC-GCaMP6f (Addgene, #73503) to hiPSC, drug screening, clone selection and myocardial differentiation were performed, and finally hiPSC-GCaMP-CMs was obtained. The cardiomyocyte ion concentration measurement system (Plexithermo, Singapore) was used to image spontaneous Ca^2+^ transients of individual hiPSC-GCaMP-CMs on an OLYMPUS IX73 microscope at 37 °C (Cell temperature control system, Plexithermo, Singapore). These data was analyzed by Image J software.

### RNA sequencing (RNA-seq)

The hiPSC-CMs were subjected to total RNA extraction, as detailed above. The concentration of qualified RNAs was determined using the QubitTM RNA Broad Range Assay kit (Thermo Fisher Scientific) on a Qubit3.0 instrument. Subsequently, stranded RNA sequencing libraries were constructed following the manufacturer’s instructions with the KCTM Stranded mRNA Library Prep Kit for Illumina® (Catalog, Wuhan, HB, China), and sequenced on a DNBSEQ-T7 sequencer (MGI Tech, Shenzhen, GD, China) using the PE150 model. The resulting raw data underwent filtering using Trimmomatic, and were then mapped to the Homo_sapiens.GRCh38 reference genome using STAR software. Read counts for each gene were obtained using featureCounts (Bioconductor). Differential expression analysis was performed using the edgeR package with a p-value cutoff of 0.05 and fold-change cutoff of 2. Functional enrichment analysis of the differentially expressed genes (DEGs) was carried out using GO and KEGG in KOBAS software. Finally, expression heatmaps were generated using the pheatmap package in R software.

### Starvation assays

Starvation assays were performed to explore the role of autophagy in hiPSC-CMs. The hiPSC-CMs were washed three times with PBS and incubated in Earle’s Balanced Salt Solution (117.24 mM NaCl, 5.33 mM KCl, 26.19 mM NaHCO3, 1.01 mM NaH2PO4, 5.56 mM D-Glucose) (Beyotime, Shanghai, China) at 37 °C for 1, 2, or 4 h. And Chloroquine (10uM, Selleck Chemicals, Houston, TX, US) was added to the starvation medium or normal medium.

### Construction of PLEKHM2-WT overexpression plasmid and production of lentivirus (LV)

WT PLEKHM2 cDNA (NM_015164.4) was subcloned into pCDH-CMV (Addgene #72266) vector without fluorescent label. Subsequently, the constructed pCDH-CMV-PLEKHM2-WT plasmid was co-transfected with psPAX (Addgene #12260) and pMD2.G (Addgene #12259) into 293 T cells. After 6 hours, we changed the medium to complete culture medium. We collected cell supernatants rich in lentivirus particles after 48 and 72 hours of cultivation and concentrated the virus supernatant by centrifugation. The viral titer was measured by quantitative PCR method.

### Infection of Ad-mRFP-EGFP-LC3 and pLV-PLEKHM2

hiPSC-CMs were seeded at a density of 10000 cells per well on 24-well plates coated with Matrigel, and cultured in fresh medium daily. On day 7 after seeding, the cells were infected with Ad-mRFP-EGFP-LC3 (Jikai gene,Shanghai, China) at a MOI of 5. On day 3 post-infection, the cells were imaged under a fluorescence microscope, and the data were analyzed using ImageJ software. pLV-PLEKHM2-WT was transfected into PLEKHM2-KO hiPSCs at a MOI of 5, and 1 week after transfection, the autophagy flux, mitochondrial function and myocardial contractility was detected.

### Western blots

The hiPSC-CMs were digested and centrifuged, and then resuspended using RIPA buffer (Beyotime, Shanghai, China) with added with a phosphatase inhibitor (Beyotime) and protease inhibitor (Beyotime). The resulting samples were incubated on ice for 30 minutes with shaking every 10 minutes, followed by centrifugation at 12,000 rpm for 15 minutes. Protein concentration was measured using the bicinchoninic acid (BCA) (Thermo Fisher Scientific) method. Equal amounts of protein were separated by gel electrophoresis and transferred to a polyvinylidene fluoride (PVDF) membrane. The membrane was blocked with 5% skim milk powder at 37°C for 1 hour and then incubated with the primary antibodies at 4 °C overnight and secondary antibodies at room temperature for 1 h. The ECL chemiluminescence system (Thermo Fisher Scientific) was used for signal amplification, and the bands were detected using the Tanon 5200 multi (Tanon, Shanghai,China). The signal intensity of the bands was analyzed and quantified using ImageJ.

### Transmission electron microscopy

The collected cells were fixed in 2.5% glutaraldehyde fixative (Servicebio, China) for 30 minutes, followed by PBS cleaning and wrapping in 1% agarose. Then, fix at room temperature with 1% osmium acid in the dark for 2 hours, and sequentially add 30%, 50%, 70%, 80%, 95%, 100%, and 100% alcohol to dehydrate for 20 minutes each time, and 100% acetone twice, each time for 15 minutes. Then, after penetration embedding, remove the resin block and slice it into ultra-thin sections using an ultra-thin slicer (Leica UC7, Leica). The slices are then placed in a copper mesh box and dried overnight at room temperature. Finally, we observed and photographed under a transmission electron microscope (HT7800/HT7700, HITACHI).

### Drug treatments

hiPSC-CMs were intermittently exposed Lipopolysaccharide (LPS) (1 ug/ml, Sigma, St. Louis, MO,US) every 6 hours per day for 1 week, observing the long-term effects of oxidative stress on WT or PLEKHM2-KO hiPSC-CMs at day 40 post myocardial differentiation. The effect of inhibiting oxidative stress on PLEKHM2-KO hiPSC-CMs was examined by treating with glutathione (GSH) (2 mM, Sangon Biotech, Shanghai, China) for 10 days, and the culture medium with GSH was changed daily until day 40 post myocardial differentiation. Rapamycin (RAPA) (500 nM, Selleck Chemicals, Houston, TX, US) was used to inhibit the activation of the mTORC1 signaling pathway for 72 hours, and evaluating the effects of RAPA on autophagic flux, mitochondrial function, and myocardial contractility in WT or PLEKHM2-KO hiPSCs-CMS at D40 post myocardial differentiation.

### Data analysis and statistics

The sample size estimation was performed using PASS 11 software, with a power of 0.9, a significance level of 5%, and the positive event rate was determined through preliminary experiments. Data was represented as mean ± standard deviation (SD). Student’s t test was used to evaluate the statistical significance of the differences between two groups. Pearson’s chi squared test was applied to evaluate statistical differences of categorical variable. One-way ANOVA or two-way ANOVA were conducted to compare differences between multiple groups. All statistical analysis and plotting were performed using Statistical Product and Service Solutions (SPSS, version 25.0) and GraphPad Prism 9.0. *p < 0.05; **p < 0.01; ***p < 0.001; ****p < 0.0001.

### Supplementary information

Supplemental Material

Original western bolts data

Reproducibility Checklist

## Data Availability

The RNA-seq raw data have been uploaded to the National Center for Biotechnology Information (NCBI) archives series (accession no.: PRJNA982145).
