# Supplementary material for: PLEKHM2 deficiency induces impaired mitochondrial clearance and elevated ROS levels in human iPSC-derived cardiomyocytes
Source: Cell Death Discov. 2024 Mar 15;10:142. doi: 10.1038/s41420-024-01907-6 (PMC10942999; doi:10.1038/s41420-024-01907-6)
Supplement: Supplementary file 1 — Supplemental Material [file 41420_2024_1907_MOESM1_ESM.docx]

**PLEKHM2 deficiency induces impaired mitochondrial clearance and elevated ROS level in human iPSC[-derived cardiomyocytes](https://insight.jci.org/articles/view/155640)**

Jianchao Zhang^1,2^**^†^**, Ying Peng^1,2^**^†^**, Wanrong Fu^1,2^**^†^**, Ruifei Wang^1,2,^**^3^**, Jinhua Cao^1,2^, Shuang Li^1,2,4^, , Xiaoxu Tian^1,2^, Zhonggen Li^1,2^, Chongpei Hua^1,2^, Yafei Zhai^1,2^, Yangyang Liu^1,2^, Mengduan Liu^1,2^, Jihong Sun^1,2^, Xiaowei Li^1,2^*, Xiaoyan Zhao^1,2^*, Jianzeng Dong^1,2,5^*

^1^Department of Cardiology, The First Affiliated Hospital of Zhengzhou University, Zhengzhou 450052, China.

^2^Henan Key Laboratory of Hereditary Cardiovascular Diseases, Zhengzhou 450052, China.

^3^Department of Cardiology, The Second Affiliated Hospital of Zhengzhou University, Zhengzhou 450052, China

^4^School of Life Sciences, Zhengzhou University, Zhengzhou 450001, Henan, China

^5^Department of Cardiology, Beijing Anzhen Hospital, Capital Medical University, National Clinical Research Centre for Cardiovascular Diseases, No. 2 Beijing Anzhen Road, Chaoyang District, Beijing 100029, China

**
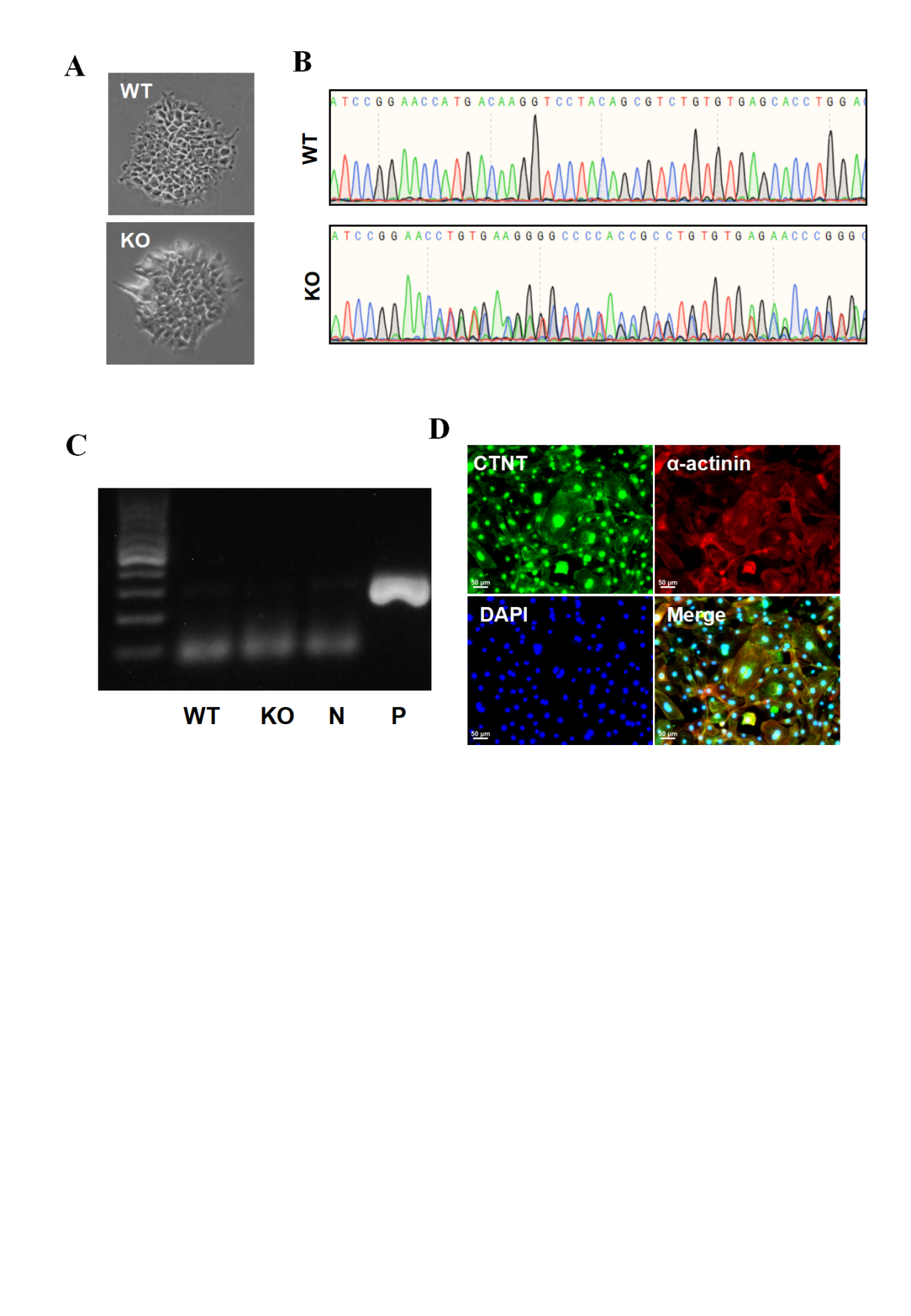
**

**Supplementary Figure 1: Characterization of the PLEKH2-KO line and and hiPSC-CM differentiation.**

(A and B) Phase image and DNA Sanger sequencing analysis of WT hiPSC and PLEKHM2-KO hiPSC. (C) Mycoplasma detection of WT and PLEKHM2-KO iPSCs. N: negative control; P: positive control. D. Representative immunofluorescence staining of sarcomeric cTnT (green) and α-actinin (red) in hiPSC-CM at day 20.


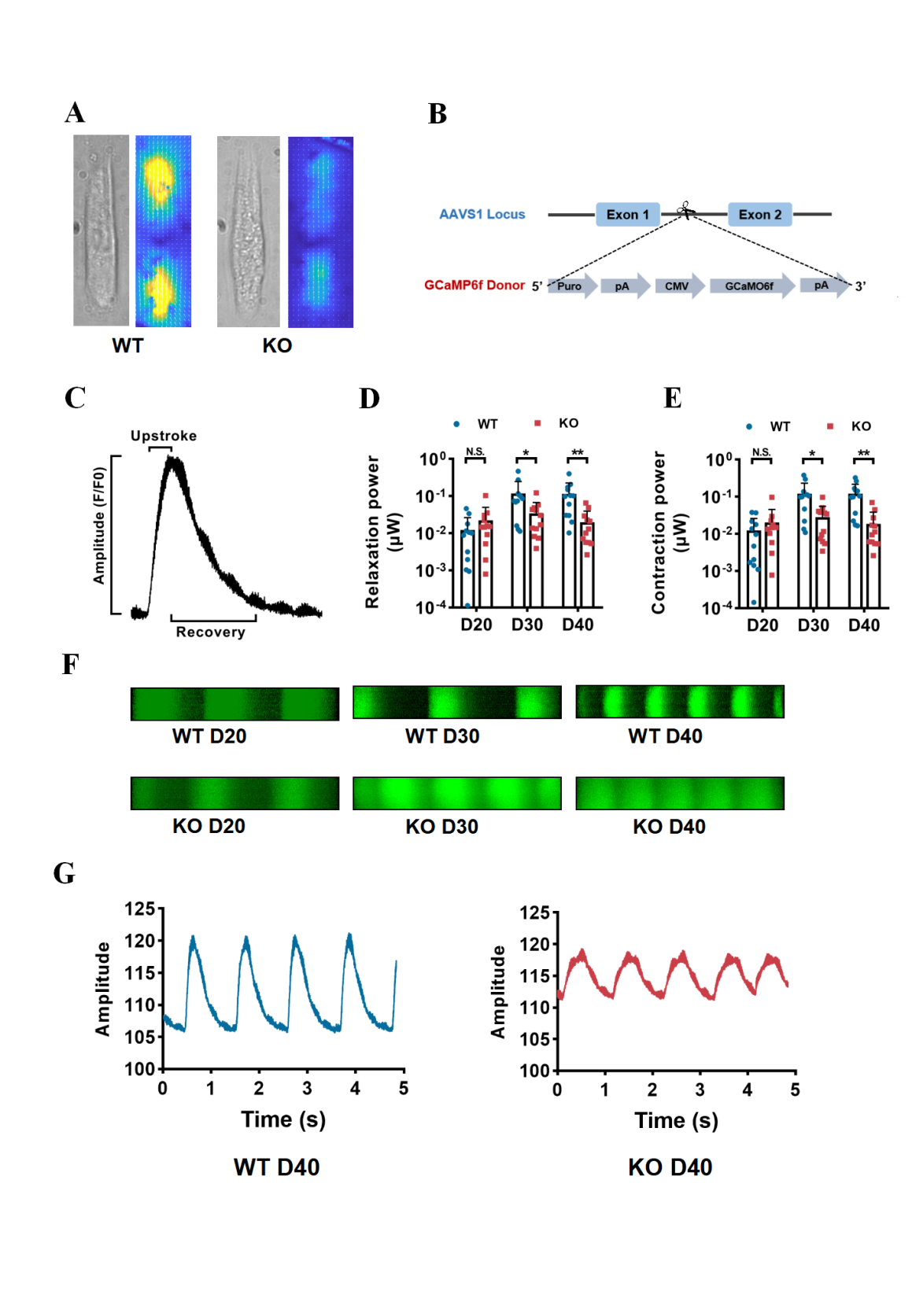


**Supplementary Figure 2: PLEKHM2 deficiency impairs myocardial contractility and calcium homeostasis.**

1. Representative images of myocardial contractility detection platform with bright field (left) and fluorescence field (right). The fluorescent beads attached to the cardiomyocytes undergo displacement as the cardiomyocytes contract, reflecting the changes in myocardial contractility. (B) Schematic of the GCaMP-expression cassette integrated into AAVS1 of WT and PLEKHM2-KO hiPSCs. (C) Parameters of calcium handling measured from space-averaged calcium transients. (D and E) Quantification of contraction and relaxation power in WT and PLEKHM2-KO hiPSC-CMs (n = 12 cells per group). Data are shown as mean ± SD. **p* < 0.05; ***p* < 0.01; ****p* < 0.001; *****p* < 0.0001; N.S., not significant, unpaired two-sided Student’s t test. (F) Representative line scan images of calcium transients in WT and PLEKHM2-KO hiPSC-CMs at days 20, 30, and 40. (G) Representative line scan images of calcium transients suggested a eleveted diastolic Ca^2+^ concentration in PLEKHM2-KO hiPSCs-CMs compared to WT hiPSCs-CMs at day 40.


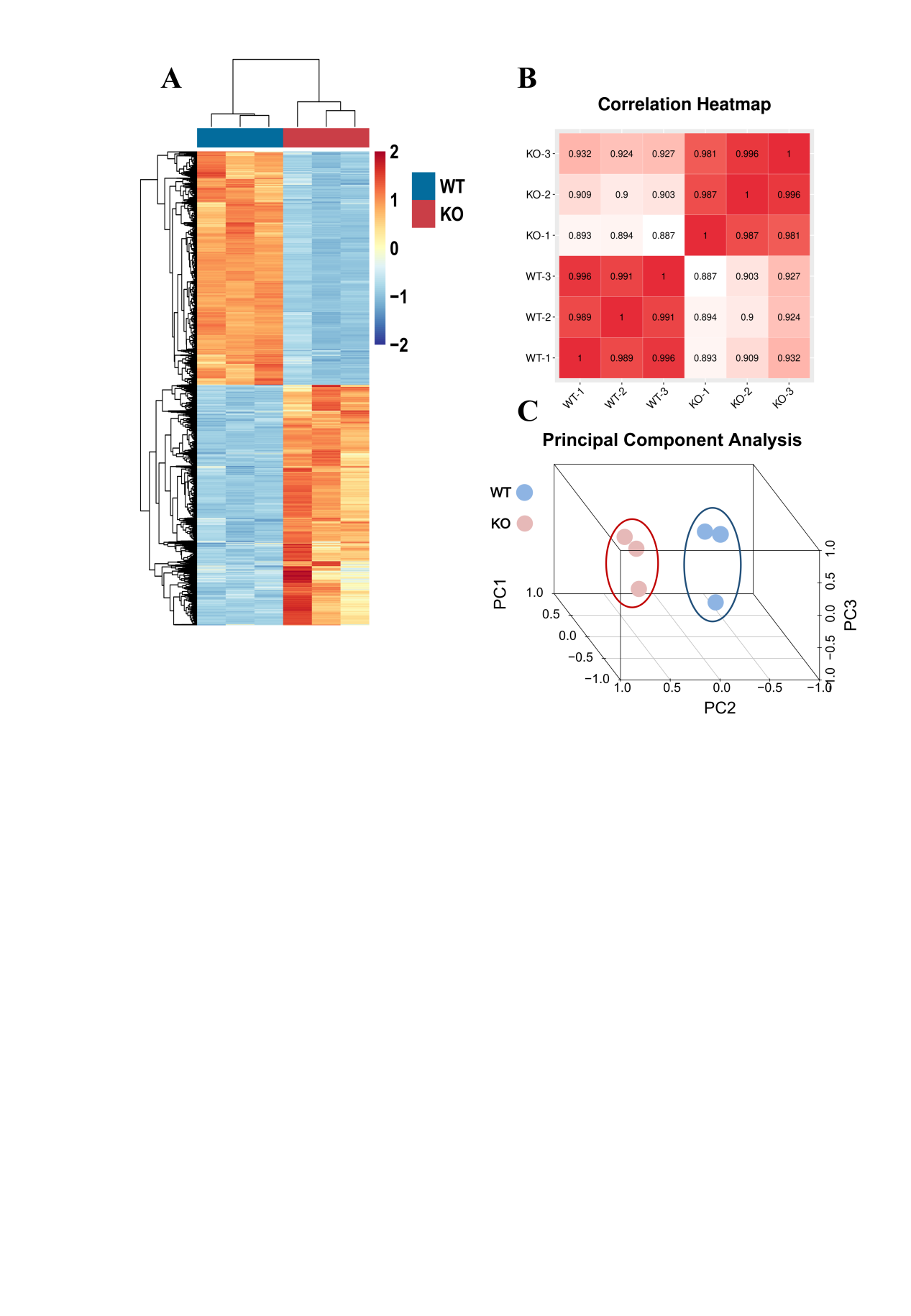


**Supplementary Figure 3: RNA sequencing of PLEKHM2-KO and WT hiPSC-CMs at day 40.**

(A) Heatmap of PLEKHM2-KO and WT hiPSC-CMs at day 40. (B) Sample correlation analysis showed significant differences between RNA sequencing sample groups and high similarity within groups. (C) The hierarchical clustering heatmap displayed inter-group dispersion and intra-group aggregation characteristics.

**
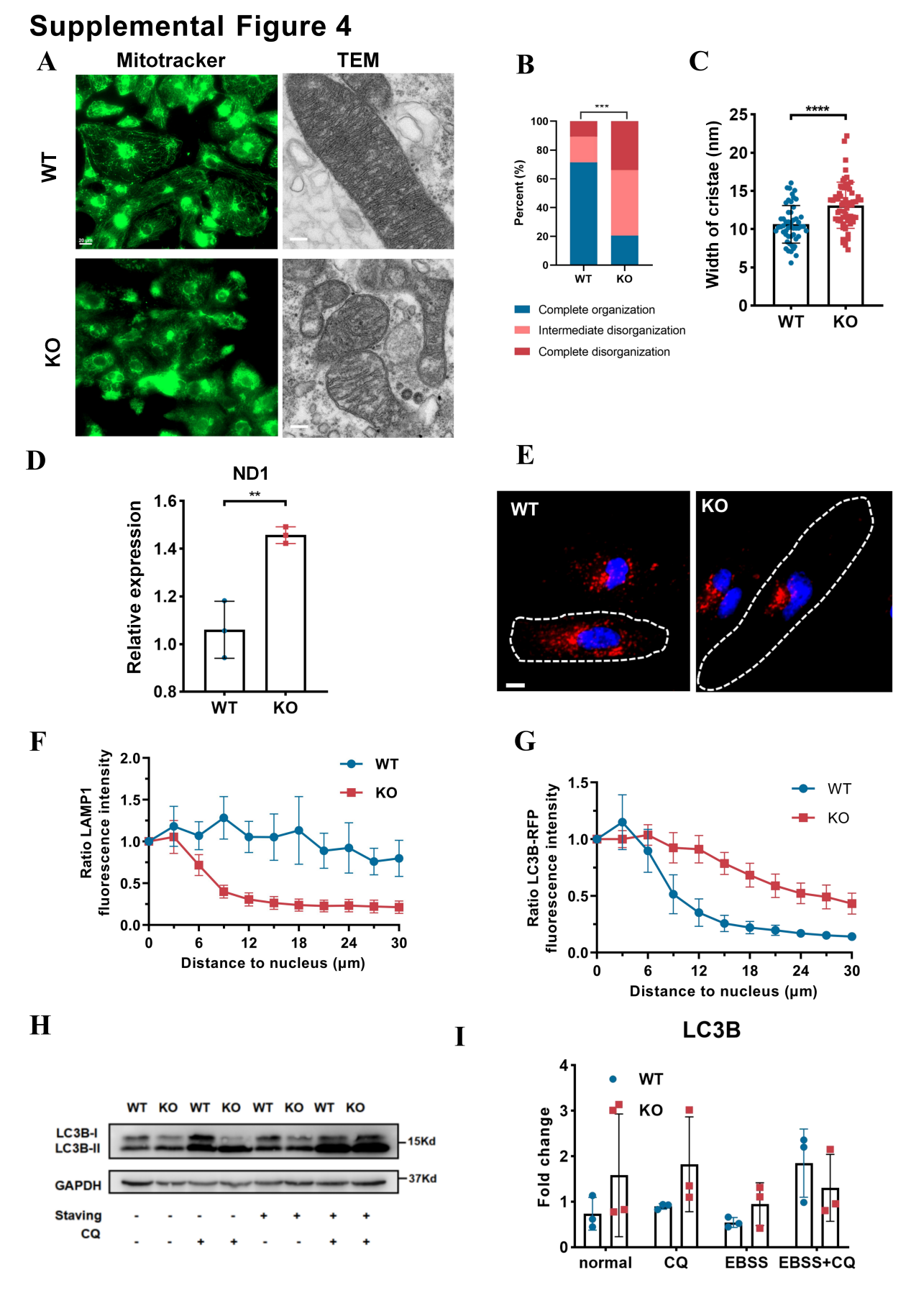
**

**Supplementary Figure 4：PLEKHM2 deficiency causes mitochondrial structural disorder, lysosomal mislocalization and impairs autophagic flux.**

1. Representative mitotraker immunofluorescence staining and transmission electron microscope (TEM) of mitochondrial. (B and C) Quantify mitochondrial morphology based on Mitotraker results and quantify mitochondrial ridge width using TEM, which indicated a significant increase in the proportion of PLEKHM2-KO hiPSC-CMs with mitochondrial structural disorder. Scale bars, 20 μm for mitotraker and 200 nm for TEM. (D) Quantification of mitochondrial copy number in hiPSC-CMs at D40 after differentiation. (E and F) Representative immunofluorescence staining and quantification of lysosomal marker LAMP1 revealed lysosomal mislocalization and aggregation around the nucleus in PLEKHM2-KO hiPSC-CMs (n = 12 cells per group). Scale bars, 10 μm. Data are shown as mean ± SD. (G) After 4 hours of starvation, LC3B-RFP puncta were still dispersed in the cytoplasm of PLEKHM2-KO hiPSC-CMs, while LC3B-RFP puncta were aggregated around the nucleus in WT group. (H and I) Quantitative WB results of LC3B II and LC3B I expression between PLEKHM2-KO and WT hiPSC-CMs (n=3). CQ: chloroquine. **p* < 0.05; ***p* < 0.01; ****p* < 0.001; **** *p* < 0.0001; N.S., not significant.


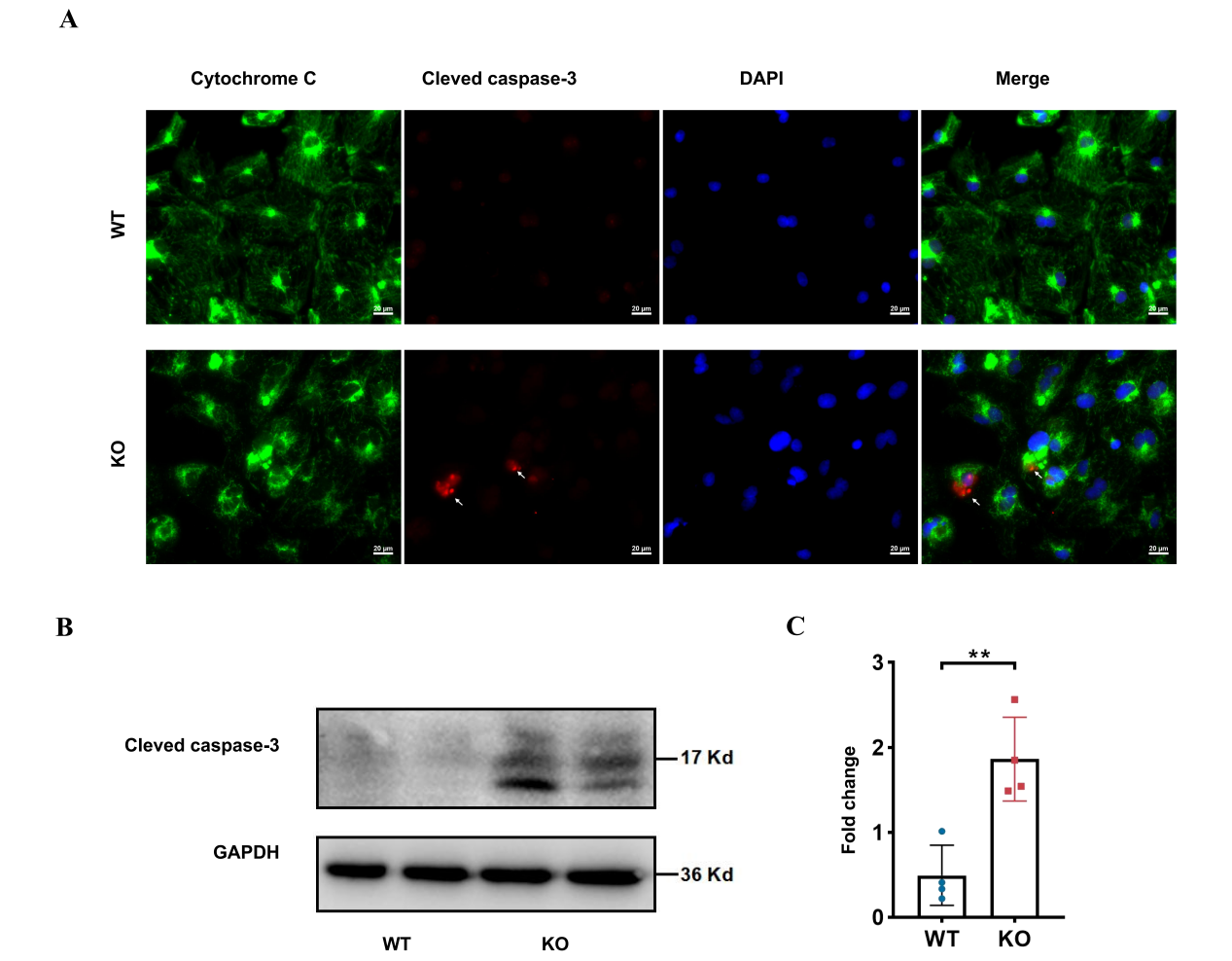


**Supplementary Figure 5: PLEKHM2 deficiency leads to ROS elevation and mitochondrial apoptosis program activation.**

(A) Representative immunofluorescence of cytochrome C (green fluorescence) and cleaved caspase-3 (red fluorescence) indicated that PLEKHM2-KO hiPSC-CMs exhibited obvious cytochrome C scattered distribution and activated cleaved caspase-3. Scale bars, 10 μm. (B and C) Representative WB and quantitative analysis of cleaved caspase-3 expression in PLEKHM2 and WT hiPSC-CMs, indicating that PLEKHM2 deficiency leads to increased cleaved caspse-3 expression and apoptosis program activation. Data are shown as mean ± SD of three independent experiments (n=4). **p* < 0.05; ***p* < 0.01; ****p* < 0.001; **** *p* < 0.0001; N.S., not significant.


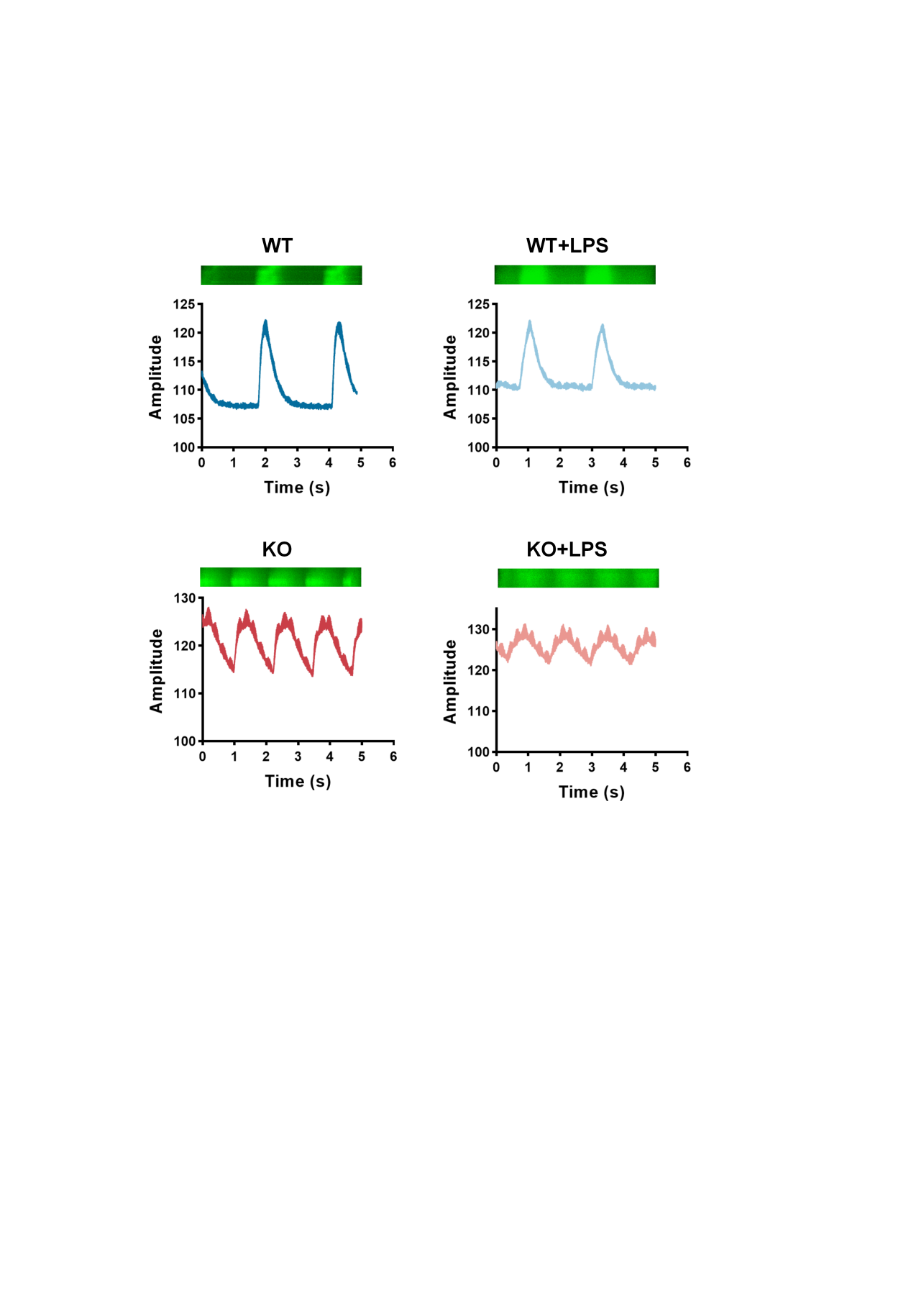


**Supplementary Figure 6:** Representative line scan images of calcium transients suggested a eleveted diastolic Ca^2+^ concentration in both PLEKHM2-KO and WT hiPSCs-CMs after LPS treatments.

**
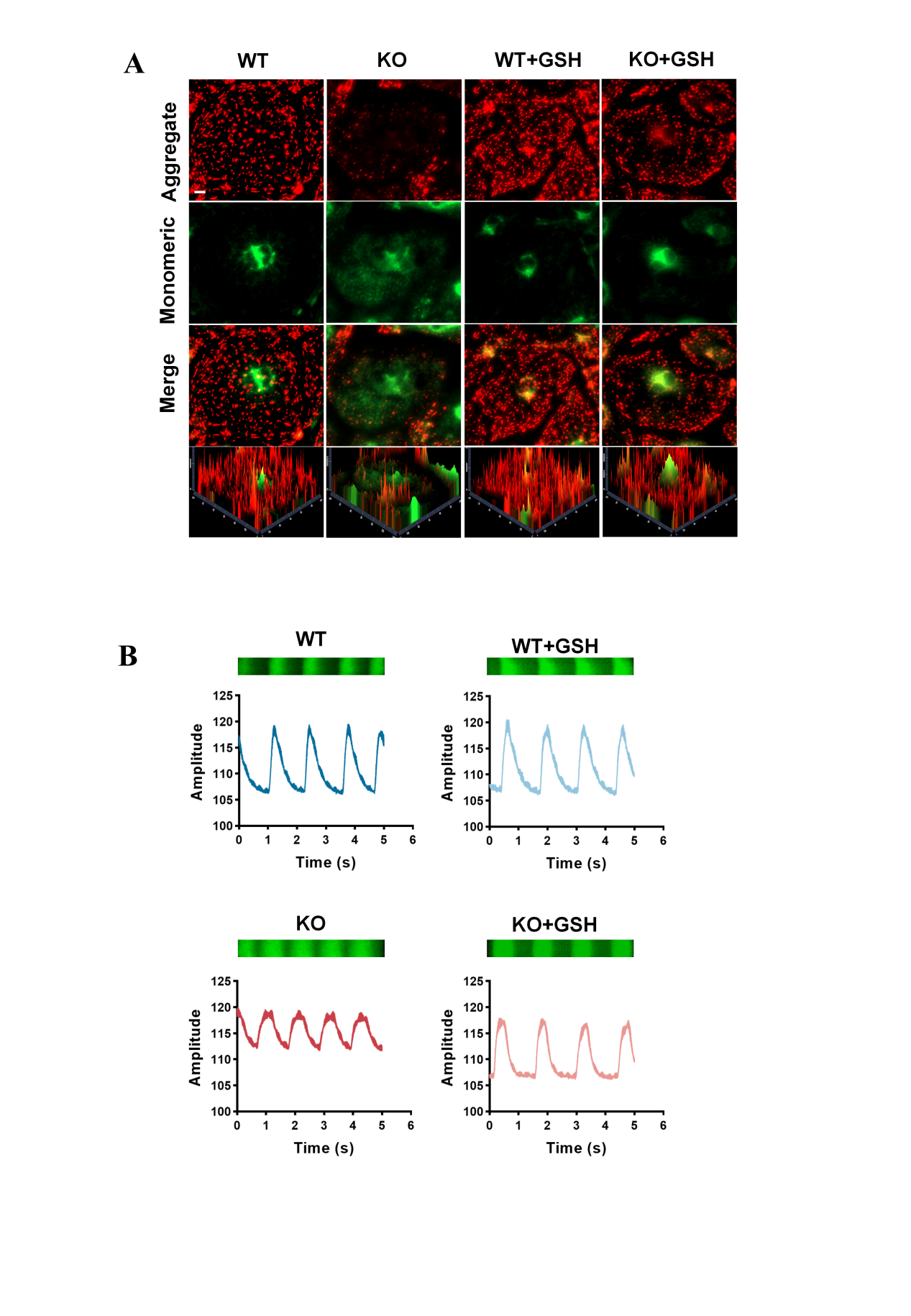
**

**Supplementary Figure 7: Treatment with GSH ameliorated PKEKHM2 deficiency-induced mitochondrial dysfunction and calcium handling abnormalities.**

(A)Representative immunofluorescence staining of *JC-1* revealed that GSH treatment ameliorated Δψm of PLEKHM2-KO hiPSCs-CMs (more than 120 cells per group). Scale bars, 10 μm. (B) Representative line scan images of calcium transients suggested a decreased diastolic Ca^2+^ concentration in PLEKHM2-KO hiPSCs-CMs after GSH treatment.


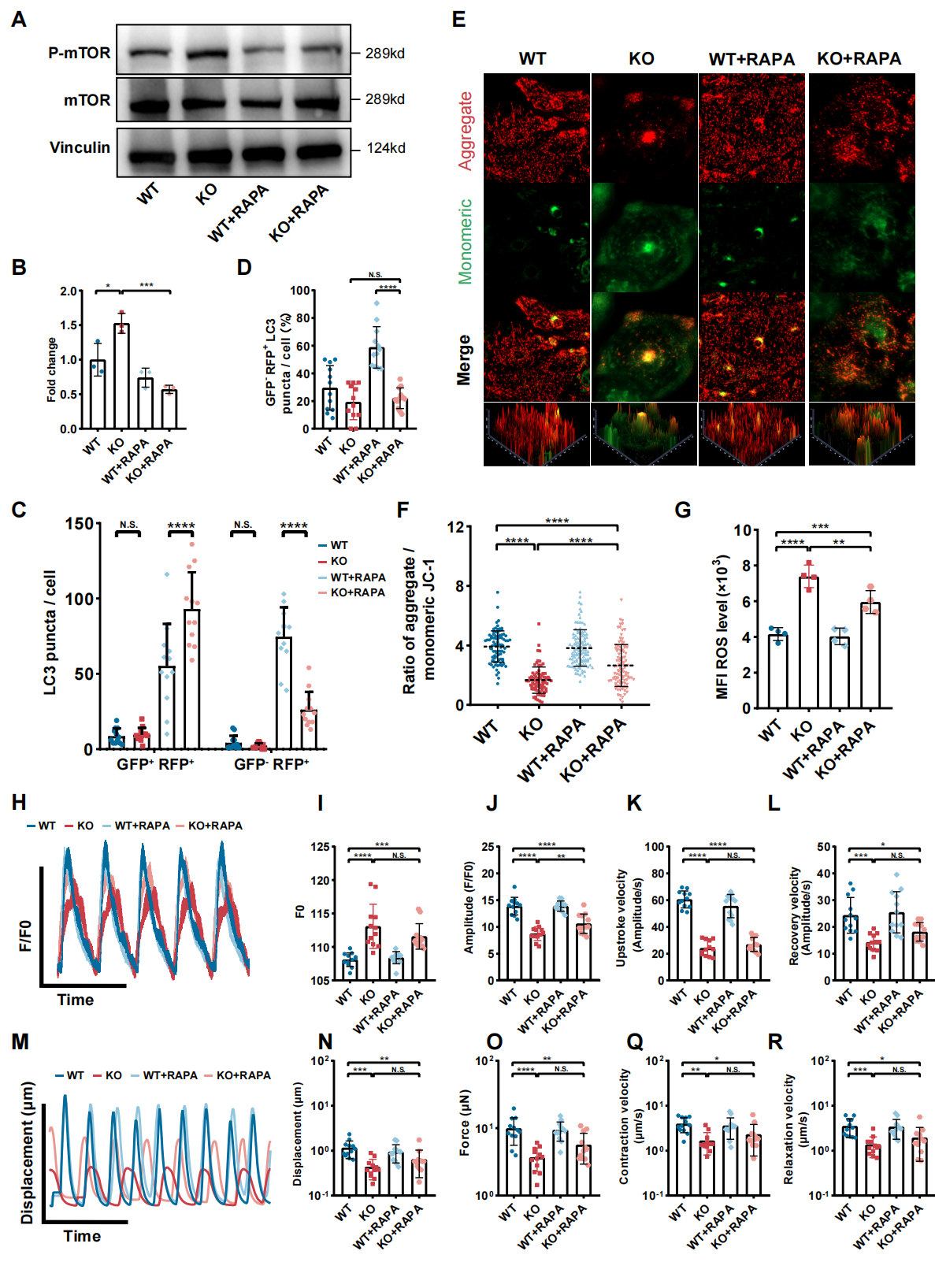


**Supplementary Figure 8: Rapamycin partially improves the phenotype of PLEKHM2-deficient cardiomyopathy.**

(A and B) Representative WB and quantitative analysis of p-mTOR in PLEKHM2-KO and WT hiPSC-CMs, indicating that PLEKHM2-KO hiPSCs-CMs deficiency activates the mTOR signaling pathway, and administration of rapamycin (RAPA) can inhibit the mTOR signaling pathway. (C and D) mRFP-EGFP-LC3 adenovirus was used to evaluate the effect of rapamycin on the autophagic flow of PLEKHM2-KO hiPSCs-CMs at day 40. 12 cells per cell line per condition were analyzed. (E and F) Representative immunofluorescence staining and and quantitative analysis of *JC-1* revealed that rapamycin treatment ameliorated Δψm of PLEKHM2-KO hiPSCs-CMs (more than 70 cells per group). Scale bars, 10 μm. (G) Quantification of cellular ROS levels showed that treatment with rapamycin can partially reduce ROS levels in PLEKHM2-KO hiPSCs-CMs at day 40 post myocardial differentiation (n = 4 independent experiments). (H) Representative line scan images of calcium transients of WT hiPSC-CMs and PLEKHM2-KO hiPSC-CMs with or without rapamycin treatment at day 40. (I-L) Quantification of amplitude, diastolic Ca^2+^ concentration, upstroke and recovery velocity in WT and PLEKHM2-KO hiPSC-CMs with or without rapamycin treatment at day 40 (n = 12 cells per group). (M) Representative line scan images of myocardial contractility in WT hiPSC-CMs and PLEKHM2-KO hiPSC-CMs with or without rapamycin treatment at day 40. (N-R), Quantification of displacement, force, contraction and relaxation velocity in WT and PLEKHM2-KO hiPSC-CMs with or without rapamycin treatment at day 40 (n = 12 cells per group). Data are shown as mean ± SD. *p < 0.05; **p < 0.01; ***p < 0.001; ****p < 0.0001; N.S., not significant.


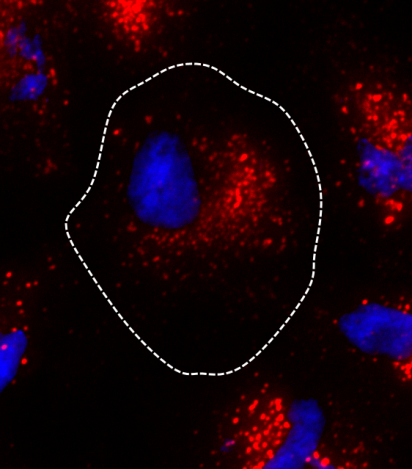

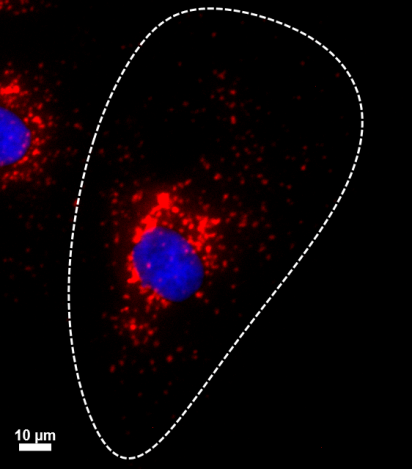

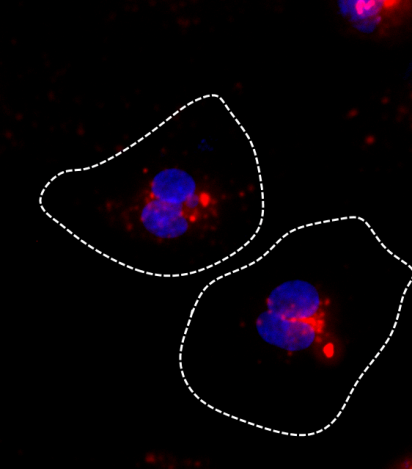


**WT**

**KO**

**WT+PLEKHM2**

**Supplementary Figure 9:** Representative immunofluorescence staining of lysosomal marker LAMP1 revealed lysosomal mislocalization and aggregation around the nucleus in PLEKHM2-KO hiPSC-CMs, and overexpression of PLEKHM2-WT can restore normal localization of lysosomes in PLEKHM2-KO hiPSC-CMs.

**Table S1：Primer sequences used for q-PCR**

| Gene | Forward 5’-3’ | Reverse 5’-3’ |
| --- | --- | --- |
| FUNDC2P2 | GAATCTGGACCTTCAGCA | GCAGCCAACTTTCCAACC |
| TFEB | CCTGGAGATGACCAACAAGC | TAGGCAGCTCCTGCTTCACC |
| CFLAR | AGTGAGGCGATTTGACCTGC | CCTCACCAATCTCTGCCATC |
| BIRC2 | CAGACACATGCAGCTCGAAT | CACCTCAAGCCACCATCACA |
| BID | TGGGACACTGTGAACCAGGA | GAGGAAGCCAAACACCAGTA |
| ENDOG | CACCTCAACCAGAATGCCTG | CACCTCAACCAGAATGCCTG |
| TSPO | TCCTACCTGGTCTGGAAAGA | CCAGCAGGAGATCCACCAAG |
| BOK | ACGCCTGGCTGAGGTGTGCG | AGGAACGCATCGGTCACCAC |
| BNIP3 | TCAGCATGAGGAACACGAGCGT | GAGGTTGTCAGACGCCTTCCAA |
| PINK1 | GTGGACCATCTGGTTCAACAGG | GCAGCCAAAATCTGCGATCACC |
| BECN1 | CTGGACACTCAGCTCAACGTCA | CTCTAGTGCCAGCTCCTTTAGC |
| BAX | TCAGGATGCGTCCACCAAGAAG | TGTGTCCACGGCGGCAATCATC |
| COX4I1 | TCGGTTTCACCGCGCTCGTTAT | TGTCCAGCATCCTCTTGGTCTG |
| TP53 | CCTCAGCATCTTATCCGAGTGG | TGGATGGTGGTACAGTCAGAGC |
| BCL2 | ATCGCCCTGTGGATGACTGAGT | GCCAGGAGAAATCAAACAGAGGC |
| DNM1L | GATGCCATAGTTGAAGTGGTGAC | CCACAAGCATCAGCAAAGTCTGG |
| OPA1 | GTGGTTGGAGATCAGAGTGCTG | GAGGACCTTCACTCAGAGTCAC |
| MFN1 | GGTGAATGAGCGGCTTTCCAAG | TCCTCCACCAAGAAATGCAGGC |
| RYR2 | AGAACTTACACACGCGACCTG | CATCTCTAACCGGACCATACTGC |
| MYH7 | GGAGTTCACACGCCTCAAAGAG | TCCTCAGCATCTGCCAGGTTGT |
| MYH6 | GGAAGACAAGGTCAACAGCCTG | TCCAGTTTCCGCTTTGCTCGCT |
| NPPA | ACAATGCCGTGTCCAACGCAGA | CTTCATTCGGCTCACTGAGCAC |
| NPPB | TCTGGCTGCTTTGGGAGGAAGA | CCTTGTGGAATCAGAAGCAGGTG |
| GAPDH | GGAGCGAGATCCCTCCAAAAT | GGCTGTTGTCATACTTCTCATGG |
| ATP2A2 | GATCACACCGCTGAATCTG | AGTATTGCGGGTTGTTCCAG |
| MT ND1 | GGCTATATACAACTACGCAAAGGC | GGTAGATGTGGCGGGTTTTAGG |

**Table S2：Primary and Secondary Antibodies**

| Type | Antibody | Application | Dilution | Species | Manufacturer  And Catalog  Number |
| --- | --- | --- | --- | --- | --- |
| Primary | PLEKHM2 Polyclonal antibody | Western blot | 1:1000 | Rabbit  Polyclonal | [Novus Biologicals](https://www.novusbio.com/products/plekhm2-antibody_nbp1-77089)  [NBP1-77089](https://www.novusbio.com/products/plekhm2-antibody_nbp1-77089) |
|  | Anti-cleved-caspase-3 | Immunofluorescence & Western blot | 1:100 & 1:1000 | Rabbit  Monoclonal | [Cell Signaling Technology](https://www.cellsignal.cn/)  #9664 |
|  | Anti-cTnT | Immunofluorescence | 1:100 | Mouse  Monoclonal | Abcam  Ab 209813 |
|  | [Anti-Cytochrome C](https://www.abcam.cn/products/primary-antibodies/cytochrome-c-antibody-epr1327-ab133504.html) | Immunofluorescence | 1:100 | Rabbit  Monoclonal | Santa Cruz  sc-13561 |
|  | Anti-α-Actinin | Immunofluorescence | 1:100 | Rabbit Polyclonal | Abcam  Ab137346 |
|  | Anti-mTOR | Western blot | 1:1000 | Rabbit  Monoclonal | [Cell Signaling Technology](https://www.cellsignal.cn/)  #2983 |
|  | Anti-p-mTOR | Western blot | 1:1000 | Rabbit  Monoclonal | [Cell Signaling Technology](https://www.cellsignal.cn/)  #2971 |
|  | Anti-Vinculin | Western blot | 1:1000 | Mouse  Monoclonal | Proteintech 66305-1-Ig |
|  | [Anti-SQSTM1](https://www.abcam.cn/products/primary-antibodies/sqstm1--p62-antibody-epr4844-autophagosome-marker-ab109012.html) | Western blot | 1:1000 | Rabbit monoclonal | [Cell Signaling Technology](https://www.cellsignal.cn/)  #88588 |
|  | [Anti-LC3B](https://www.abcam.cn/products/primary-antibodies/lc3b-antibody-epr18709-autophagosome-marker-ab192890.html) | Western blot | 1:1000 | Rabbit monoclonal | [Cell Signaling Technology](https://www.cellsignal.cn/)  #3868 |
|  | Anti-GAPDH | Western blot | 1:1000 | Mouse  Monoclonal | Proteintech 60004-1 |
|  | Anti-OCT4 | Immunofluorescence | 1:100 | Rabbit Monoclonal | Abcam  Ab 19857 |
|  | Anti-SSEA4 | Immunofluorescence | 1:100 | Mouse  Monoclona | Invitrogen  41-4000 |
| Secondary | Goat  anti-Mouse IgG Alexa  Fluor 594 | Immunofluorescence | 1:200 | Goat  anti-Mouse  IgG | Invitrogen  A11032 |
|  | Goat  anti-Rabbit  IgG Alexa Fluor 488 | Immunofluorescence | 1:200 | Goat  anti-Rabbit  IgG | Invitrogen  A11034 |
|  | Goat anti- Mouse IgG Alexa Fluor 488 | Immunofluorescence | 1:200 | Goat  anti-Mouse  IgG | Invitrogen  A11029 |
|  | Goat  anti-Rabbit  IgG Alexa Fluor 594 | Immunofluorescence | 1:200 | Goat  anti-Rabbit  IgG | Invitrogen  A-11037 |
|  | [Goat anti-Rabbit IgG (H+L) Secondary Antibody, HRP](https://www.thermofisher.cn/antibody/product/Goat-anti-Rabbit-IgG-H-L-Secondary-Antibody-Polyclonal/31460?imageId=257486) | Western blot | 1:5000 | Goat anti-Rabbit IgG | Proteintech SA00001-1 |
|  | [Goat anti-Mouse IgG (H+L) Secondary Antibody, HRP](https://www.thermofisher.cn/antibody/product/Goat-anti-Rabbit-IgG-H-L-Secondary-Antibody-Polyclonal/31460?imageId=257486) | Western blot | 1:5000 | Goat anti-Mouse IgG | Proteintech SA00001-2 |
