# Supplementary material for: PLEKHM2 deficiency induces impaired mitochondrial clearance and elevated ROS levels in human iPSC-derived cardiomyocytes
Source: Cell Death Discov. 2024 Mar 15;10:142. doi: 10.1038/s41420-024-01907-6 (PMC10942999; doi:10.1038/s41420-024-01907-6)
Supplement: Supplementary file 2 — Original western bolts data [file 41420_2024_1907_MOESM2_ESM.docx]

**PLEKHM2 deficiency induces impaired mitochondrial clearance and elevated ROS level in human iPSC[-derived cardiomyocytes](https://insight.jci.org/articles/view/155640)**

Jianchao Zhang^1,2^**^†^**, Ying Peng^1,2^**^†^**, Wanrong Fu^1,2^**^†^**, Ruifei Wang^1,2,^**^3^**, Jinhua Cao^1,2^, Shuang Li^1,2,4^, , Xiaoxu Tian^1,2^, Zhonggen Li^1,2^, Chongpei Hua^1,2^, Yafei Zhai^1,2^, Yangyang Liu^1,2^, Mengduan Liu^1,2^, Jihong Sun^1,2^, Xiaowei Li^1,2^*, Xiaoyan Zhao^1,2^*, Jianzeng Dong^1,2,5^*

^1^Department of Cardiology, The First Affiliated Hospital of Zhengzhou University, Zhengzhou 450052, China.

^2^Henan Key Laboratory of Hereditary Cardiovascular Diseases, Zhengzhou 450052, China.

^3^Department of Cardiology, The Second Affiliated Hospital of Zhengzhou University, Zhengzhou 450052, China

^4^School of Life Sciences, Zhengzhou University, Zhengzhou 450001, Henan, China

^5^Department of Cardiology, Beijing Anzhen Hospital, Capital Medical University, National Clinical Research Centre for Cardiovascular Diseases, No. 2 Beijing Anzhen Road, Chaoyang District, Beijing 100029, China

**Western bolts data**


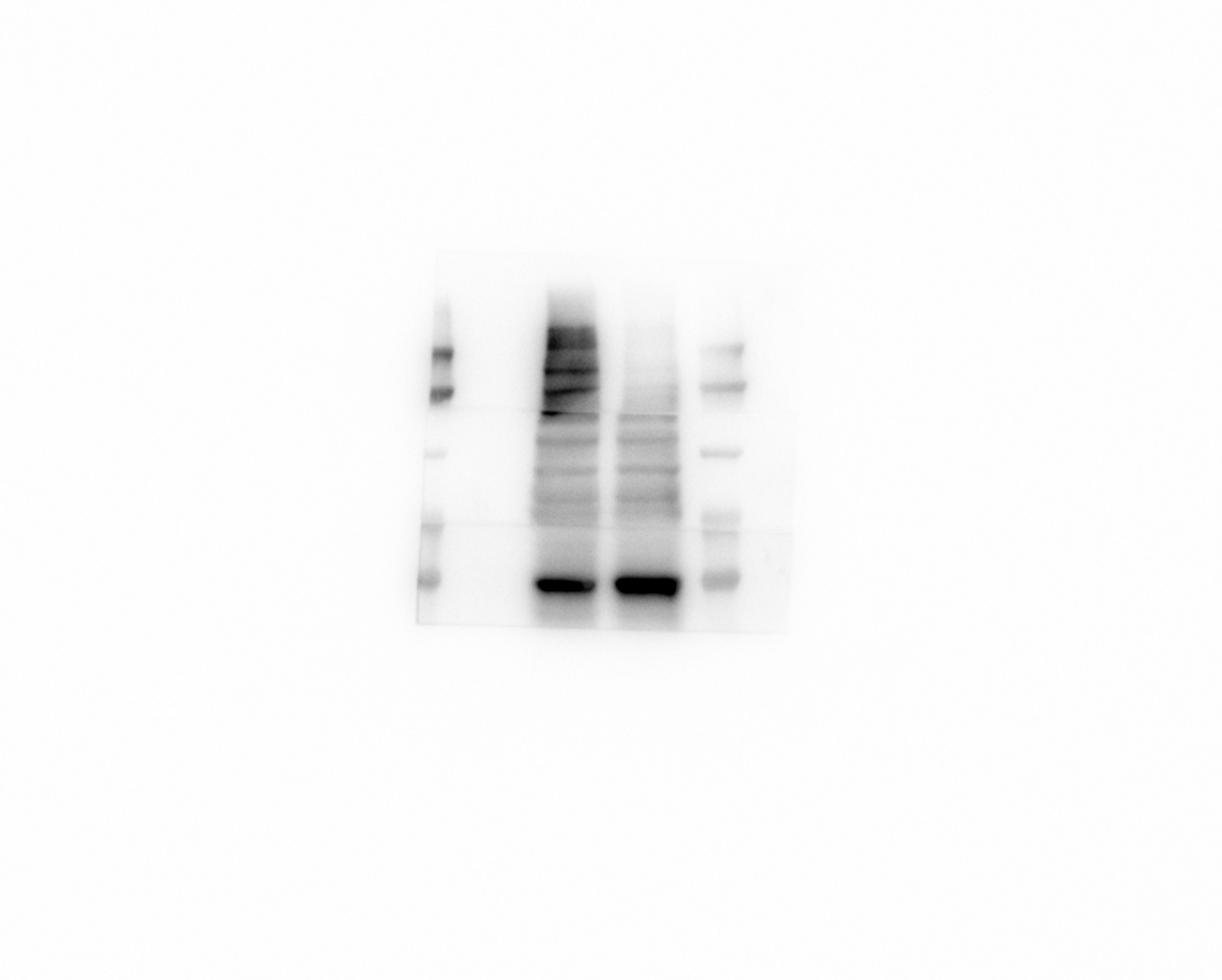


**Figure. 1D PLEKHM2 and GAPDH (hiPSC-CM of WT or PLEKHM2-KO)**


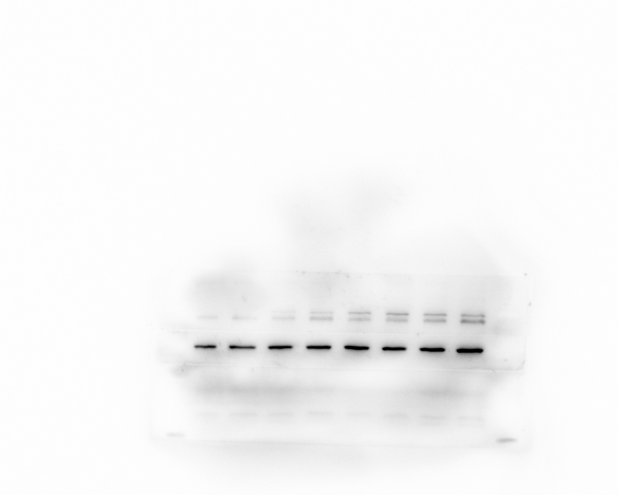


**Figure. 4G p62 and GAPDH (hiPSC-CM of WT or PLEKHM2-KO treated with EBSS or chloroquine)**


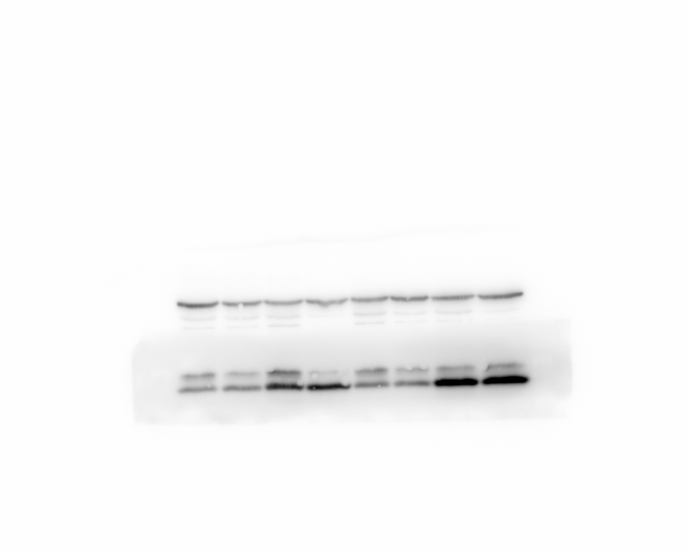


**Supplementary Figure. 4F LC3B and GAPDH (hiPSC-CM of WT or PLEKHM2-KO treated with EBSS or chloroquine)**

**
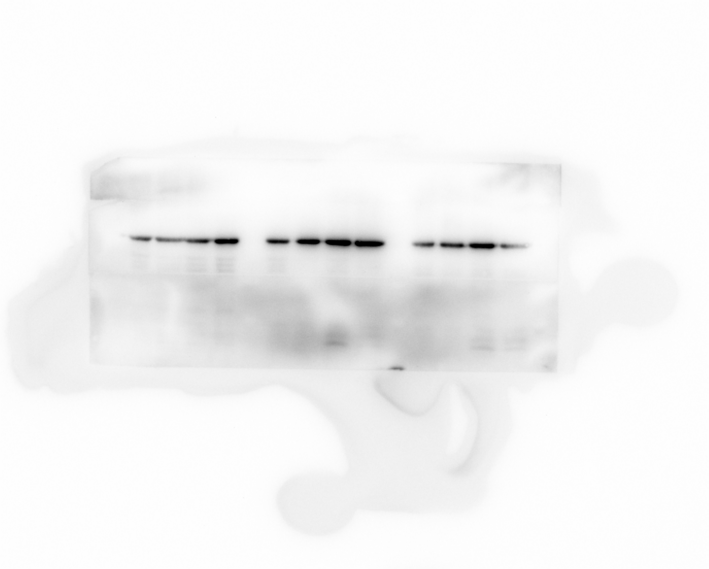
**

**Supplementary Figure. 5B cleaved caspse-3 and GAPDH (hiPSC-CM of WT or PLEKHM2-KO)**


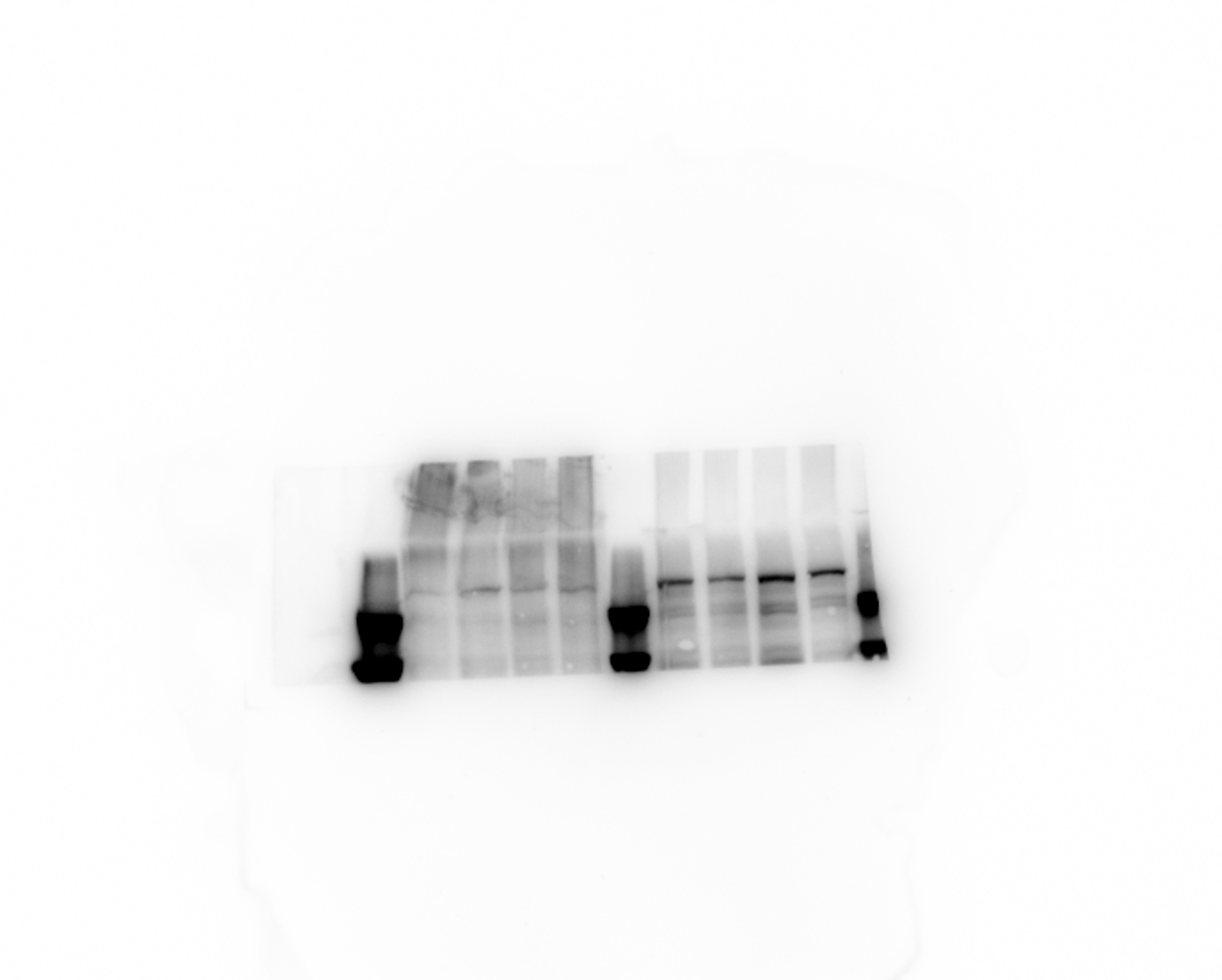


**
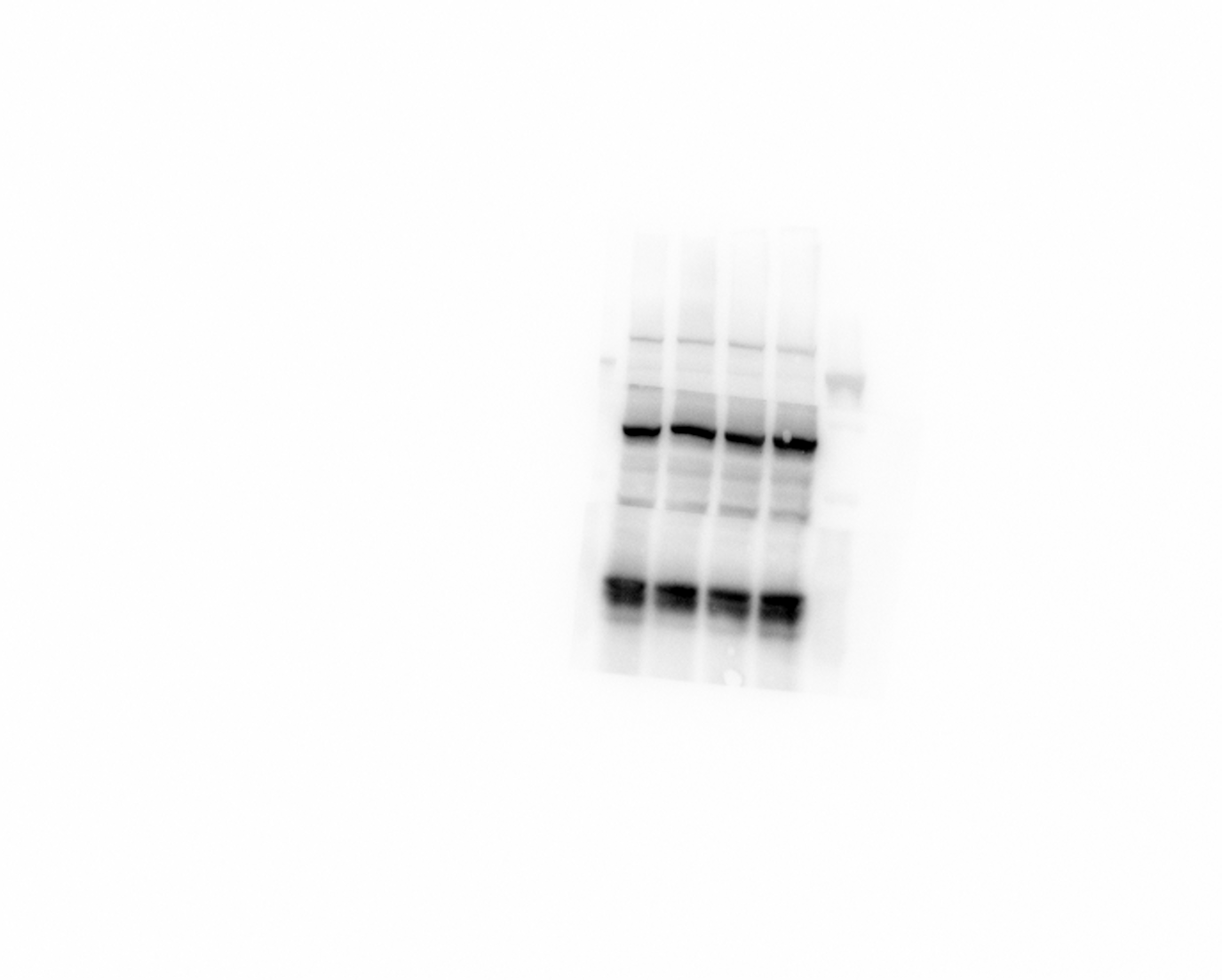
**

**Supplementary Figure. 8A mTOR, P-mTOR and vinculin ( WT and PLEKHM2-KO hiPSC-CM treated or untreated with RAPA)**
